# Multiview deep-learning-enabled histopathology for prognostic and therapeutic stratification in stage II colorectal cancer: A retrospective multicenter study

**DOI:** 10.1371/journal.pmed.1004614

**Published:** 2026-01-13

**Authors:** Zihan Zhao, Dexia Chen, Ruixuan Wang, Xinke Zhang, Xiaobo Wen, Xueyi Zheng, Shasha Liu, Hao Chen, Yuqian Zhang, Dan Huang, Chengyou Zheng, Mengke Ma, Dan Xie, Yan Sun, Xiaosheng He, Muyan Cai

**Affiliations:** 1 Department of Pathology, State Key Laboratory of Oncology in South China, Guangdong Provincial Clinical Research Center for Cancer, Sun Yat-sen University Cancer Center, Guangzhou, Guangdong, China; 2 School of Computer Science and Engineering, Sun Yat-sen University, Guangzhou, Guangdong, China; 3 Department of Pathology, Tianjin Medical University Cancer Institute and Hospital, Tianjin, China; 4 Department of General Surgery (Colorectal Surgery), The Sixth Affiliated Hospital, Sun Yat-sen University, Guangzhou, Guangdong, China; 5 Electrical Engineering & Computer Science, Johns Hopkins University, Baltimore, Maryland, United States of America; 6 Department of Pathology, Fudan University Shanghai Cancer Center, Shanghai, China; Washington University in St Louis, UNITED STATES OF AMERICA

## Abstract

**Background:**

Approximately 20% of patients with stage II colorectal cancer (CRC) experience tumor relapse despite standard surgical treatment. Histopathological analysis holds promise for postsurgical risk stratification and guiding adjuvant chemotherapy (ACT) decisions. The aim of this study was to use deep learning to extract explainable tissue biomarkers from whole-slide images.

**Methods and findings:**

In this retrospective cohort study, we developed and validated SurvFinder, an interpretable deep learning framework designed to autonomously identify tissue-based risk biomarkers from hematoxylin and eosin (H&E)-stained slides. The framework aims to support individualized risk stratification and explore associations with treatment outcomes. The present study included 6,950 H&E slides from 1,604 patients with stage II CRC across four independent cohorts in China. Patients were enrolled from 2012 to 2018 and followed for a minimum of 24 months. The primary outcome of the study was relapse-free survival (RFS). Our analyses identified tertiary lymphoid structures (TLSs) as critical prognostic features in stage II CRC. The multi-view integration of TLS characteristics by SurvFinder consistently demonstrated superior predictive and prognostic accuracy across four multicenter datasets (AUROC with 95% confidence interval [CI]: 0.827 [0.789,0.864], 0.805 [0.749,0.860], 0.805 [0.748,0.861], and 0.712 [0.621,0.804]), surpassing traditional clinical prognostic parameters (hazard ratio [HR]: 8.23, 95% CI: 5.43–12.47; *p* < 0.001). Using explainable AI (XAI) methods, we ensured model transparency and identified key TLS features-such as their location at the tumor periphery and their maturity state-as significant factors influencing prognosis and the efficacy of adjuvant therapy. The retrospective design without prospective validation and real-world clinical deployment is the main limitation of this study.

**Conclusions:**

Together, these results highlight the potential utility of deep learning-based histopathological analysis for automated risk stratification in stage II CRC. In particular, our findings support the relevance of TLSs as a histological biomarker with potential implications for personalizing ACT decisions.

## Author summary

**Why was this study done?:**

- Approximately 20% of patients with stage II colorectal cancer (CRC) experience recurrence after surgery, but it remains challenging to identify which individuals are at high risk and might benefit from adjuvant chemotherapy.
- Traditional clinicopathological markers provide some guidance but often lack sufficient accuracy for personalized treatment decisions.
- There is a critical need for novel, scalable tools that can enhance risk stratification and inform therapeutic strategies using data routinely available in clinical practice.

**What did the researchers do and find?:**

- We developed a deep learning model that automatically identifies key histological features to help predict patient risk and treatment benefit in stage II CRC.
- The model identified tertiary lymphoid structure-like features as strong prognostic markers and integrated multiple histological views to improve prediction of disease recurrence.
- The framework reliably stratified patients by relapse risk and identified subgroups that appeared to derive greater benefit from adjuvant chemotherapy.

**What do these findings mean?:**

- This study demonstrates the potential of artificial intelligence to extract clinically meaningful features from standard histological slides, offering a non-invasive and cost-effective approach to guide treatment decisions.
- Our findings support the future development of AI-assisted tools that can augment clinical decision-making with more consistent, interpretable, and personalized risk assessments in stage II CRC.
- As a retrospective multicenter study, it is subject to selection bias and confounding, limiting causal inference.

## Abstract 3

In a retrospective analysis of whole slide images from individuals with stage II colorectal cancer, used to train a deep learning model, Zihan Zhao and colleagues identify histological features associated with risk of disease recurrence.

## Introduction

Colorectal cancer (CRC) ranks as the third most common malignancy globally and the fourth leading cause of cancer-related mortality. Approximately 25% of all CRC cases diagnosed annually are classified as stage II [1]. Unfortunately, around 20% of these patients experience fatal recurrence despite undergoing surgical resection alone [2]. The National Comprehensive Cancer Network (NCCN) guidelines recommend adjuvant chemotherapy (ACT) for stage II CRC patients exhibiting high-risk clinicopathological features, such as pT4 stage, localized perforation, or insufficient lymph node sampling (fewer than 12 nodes) [3,4]. However, the efficacy of ACT in these traditionally high-risk patients is limited, and the selection of patients who would benefit from adjuvant therapies remains a considerable challenge [5]. Moreover, ACT is associated with significant risks, including a 0.5%–1% mortality rate and severe side-effects in approximately 20% of patients, alongside significant financial burdens [6–8].

In recent years, advances in genetic and molecular approaches have led to the development of prognostic biomarkers, such as DNA mismatch repair (MMR), microRNAs, and circulating tumor DNA [9–11]. These biomarkers have shown potential in improving risk stratification and guiding treatment decisions for CRC patients. However, only a small proportion of stage II CRC patients (less than 15%) are MMR-deficient, and the prognostic value of other biomarkers has not been conclusively validated in clinical practice [12,13]. Additionally, most genetic biomarkers require specialized assays that may not be readily available in all clinical settings, limiting their widespread adoption. This highlights the need for efficient, accessible prognostic and predictive biomarkers that can be derived from routine clinical data.

Hematoxylin and eosin (H&E) stained histopathological slides, which are routinely available for every CRC patient, contain a wealth of histomorphological information critical for diagnosis and prognosis. Recent advancements in digital pathology have leveraged deep learning (DL) models to H&E slides for various tasks, including diagnostic category, molecular classification, and prognostic prediction in CRC [14–16]. By directly analyzing whole-slide images (WSIs), DL models can evaluate complex histopathological pattern, contextualize them with coexisting patterns, and generate risk scores without relying on predefined image features [17].

In this study, we hypothesized that DL could robustly identify histological biomarkers that are predictive of prognosis and therapeutic benefit in stage II CRC. To address this question, we developed and validated an explainable deep-learning framework across multiple independent cohorts to uncover prognostically relevant tissue features and integrate them with clinicopathological variables for comprehensive risk stratification and informed therapeutic decision-making.

## Methods

### Study participants

All patient-related information in this study was reviewed and approved by the Institutional Ethics Committee at Sun Yat-sen University Cancer Center (Approval No. SL-B2024-229-01). As no patients were directly recruited for this study, informed consent was waived.

This retrospective study was conducted to develop SurvFinder, using data from four cohorts: three independent cohorts from separate medical centers and one cohort from the publicly available TCGA dataset. All cohorts contributed to both the development of the model and prognostic analyses. Each cohort consisted of patients with a confirmed diagnosis of stage II CRC through molecular and immunohistochemical testing of FFPE samples. For patients who received ACT in both internal and external cohorts, treatment regimens were administered according to standard protocols recommended by the NCCN guidelines [3,4]. The majority of patients were treated with fluoropyrimidine-based therapies, including: (1) monotherapy, primarily with capecitabine; (2) doublet regimens, mainly FOLFOX or XELOX; and (3) triplet regimens, typically FOLFOX or XELOX combined with monoclonal antibodies.

The Internal-CRCII cohort, used for model training and internal evaluation, included 842 cases diagnosed at Sun Yat-sen University Cancer Center between November 1, 2014, and December 31, 2018. Two external cohorts were utilized for validation: External-CRCII-1 cohort (438 cases diagnosed between August 1, 2012, and October 31, 2018, with accessible follow-up information from Tianjin Medical University Cancer Institute and Hospital) and External-CRCII-2 (405 patients diagnosed between January 1, 2015, and December 31, 2018, with available follow-up information from The Sixth Affiliated Hospital of Sun Yat-sen University). For each case in these three cohorts, clinicopathological parameters, including 14 conventional risk-related factors and the application of ACT, were collected. All tumor-related indicators (e.g., tumor budding, perineural invasion, vascular invasion, tumor differentiation, etc.) were assessed by the same experienced pathologist across all cohorts to ensure consistency.

Patients from all datasets underwent additional filtering before training based on the following exclusion criteria: (1) Patients who had received preoperative treatments, including neoadjuvant chemotherapy or radiotherapy; (2) Patients with a prior history of treatment for other cancers before the diagnosis of stage II CRC; (3) Patients with no observed relapse and a follow-up period of less than 24 months; (4) Patients lacking one or more of the 14 key clinicopathological variables used for comparison with MVNet (S7 Table); (5) Patients who died from causes unrelated to cancer; and (6) Patients with fewer than three available slides after quality control checks (e.g., slides with issues such as tissue folds, fractures, absence of tumor tissue, or those out of focus). Random sampling experiments show that the prognostic performance of the model improves as the number of included tumor slides per patient increases (S8 Table). Following the application of these exclusion criteria, the final numbers of cases in the Internal-CRCII, External-CRCII-1, and External-CRCII-2 datasets comprised 743, 352, and 331 cases, respectively.

Additionally, the study incorporated the TCGA-CRCII dataset, which comprised 229 cases from the TCGA-COAD and TCGA-READ projects. Due to lower data quality in the TCGA dataset, exclusion criteria were modified: patients without follow-up data, diagnostic slides, or slides meeting quality standards were excluded. To evaluate the generalizability of our model in cases with limited tissue sampling, no slide-number restriction to the TCGA-CRCII cohort was applied during validation. This resulted in 178 cases being used for validation.

### Digitalization, immunohistochemistry, and WSI preprocessing

For each case, all available H&E-stained tumor slides were included for further analyses. All WSIs included in this study were scanned at a magnification of 40×, corresponding to a resolution of 0.25 μm/pixel, ensuring uniform pixel size across all datasets. Slides from Internal-CRCII and External-CRCII-1 cohorts were scanned with a PHILIPS Ultra Fast Scanner (Philips Electronics N.V., Amsterdam, the Netherlands) and saved in iSyntax format, while those from External-CRCII-2 dataset were scanned using an SQS-600P scanner (Shengqiang Technology, Shenzhen, China) and stored in SVS format. The tumor slides for the TCGA-CRCII dataset were downloaded from the Genomic Data Commons portal (https://portal.gdc.cancer.gov/).

WSINet was trained without any pixel-level or region-level annotations. Only slide-level labels (relapse versus non-relapse) were used for supervision. A total of 120 slides from both Internal-CRCII and External-CRCIIs were randomly selected to annotate normal, tumor, and tertiary lymphoid structure (TLS) regions for the development of SegNet. Immunohistochemistry (IHC) staining, using CD20, CD21, and CD10 markers, was employed to identify and define TLS phenotypes [18]. An experienced pathologist delineated TLS regions based on IHC results (S11 Fig). Specifically, TLS annotations on H&E slides were manually derived by referencing consecutive IHC-stained slides (CD20, CD21, CD10), based on morphological correspondence between serial sections.

To improve annotation consistency and accuracy, a meta-annotation method was applied to the procedure of labeling tumor and normal tissues [19]. For each WSI, at least four rectangular boxes containing representative normal and tumor tissue areas were extracted. These areas included various tissue types, such as vascular, smooth muscle, necrosis, normal, and tumor glands. Two pathologists with extensive experience in CRC diagnosis participated in tissue annotation and revision process. They used the open-source software QuPath (v0.2.3) and performed the annotations blinded to patient information and the study’s purpose to minimized bias. After annotation, the WSIs were further segmented into non-overlapping 256 × 256 tissue tiles, with the white background removed using Otsu’s method. The comparative visualization results demonstrate that the Otsu’s method effectively removes the white background (S12 Fig). To account for variations in tissue color from different scanning devices, all tiles from the External-CRCII-2 cohort were color-normalized to those from the Internal-CRCII cohort using the Vahadane method [20]. The predictive performance exhibited similar trends across both stained and unstained datasets, although the stained group consistently demonstrated superior accuracy (S13 Fig).

### Deep learning model architecture

**Fig 1:**
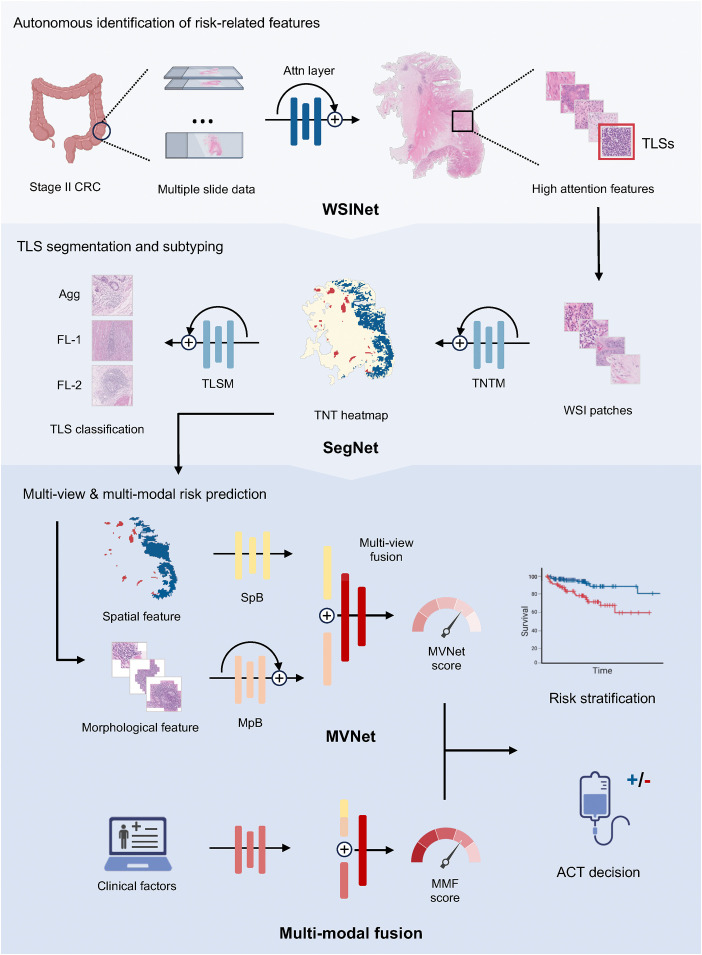
The workflow of SurvFinder. The SurvFinder framework consists of three main components: autonomous identification of risk-related tissue features, multi-class segmentation of prognostic biomarkers, and multi-view and multimodal risk prediction. WSINet is utilized to identify prognosis-associated tissue features, such as tertiary lymphoid structures (TLSs). SegNet is designed for automatic segmentation of TLSs and tumor tissue, as well as differentiation of TLS phenotypes. MVNet integrates multi-view characteristics of TLSs for prognosis evaluation and therapeutic decision-making, with multi-modal fusion also implemented. CRC, colorectal cancer; Attn, attention; TLS, tertiary lymphoid structure; SegNet, segmentation network; TNTM, TLS-normal-tumor segmentation model; TLSM, TLS subtyping model; Agg, aggregates; FL-1, primary follicles; FL-2, secondary follicles; MVNet, multi-view network; SpB, spatial feature branch; MpB, morphological feature branch; MMF, multi-modal fusion; ACT, adjuvant chemotherapy. *Created in BioRender. Oa, A. (2025)* https://BioRender.com/a45w458.

The SurvFinder framework consists of four principal neural networks: WSINet, SegNet, MVNet, and MMF (Fig 1), each designed to perform a specific task within the overall architecture.

WSINet identifies prognostic tissue biomarkers from WSIs. SegNet then segments the regions of the biomarkers identified by WSINet, generating tissue heatmaps. MVNet further processes these segmented histological biomarkers by extracting and integrating multi-view features for risk stratification. MMF combines the multi-view features from MVNet with clinical data to produce a comprehensive fusion-based prediction.

WSINet, inspired by Lu and colleagues [21], predicts patient prognosis by identifying tissue patterns within WSIs. We utilized pretrained CTransPath model, developed by Wang and colleagues [22], which leverages the Swin Transformer architecture and was trained on the TCGA and PAIP datasets. Features were extracted from all image patches in each WSI. To comprehensively capture the tumor microenvironment, each case included at least three tumor slides, aggregating patch features across all WSIs per case. These case features are then processed through a simplified attention network, which assigns attention scores to each patch. Let ${𝐫}_{\text{1}},{𝐫}_{\text{2}},\ldots ,{𝐫}_{N}\in R^{\text{768}\times N}$ denote the feature set for all *N* patches of a given case. The attention score for the i-th patch is estimated by

$S_{i}=\frac{exp(𝐖(tanh({𝐖}_{a}\cdot {𝐟}_{i})⊙\sigma ({𝐖}_{b}\cdot {𝐟}_{i})))}{\sum_{j=\text{1}}^{N}\;\;exp(𝐖(tanh({𝐖}_{a}\cdot {𝐟}_{j})⊙\sigma ({𝐖}_{b}\cdot {𝐟}_{j})))}$

where 𝐖, ${𝐖}_{a}$, and ${𝐖}_{b}$ are trainable weight matrices, and ${𝐟}_{i}$ is the feature vector of the i-th patch, obtained by passing ${𝐫}_{i}$ through two fully connected layers. tanh(·) and σ(·) represent the hyperbolic tangent and sigmoid activation functions, respectively. ⊙ denotes element-wise multiplication. $S_{i}$ quantifies the relative contribution of the i-th patch to the overall prognostic outcome for the case. Using these scores, we perform a weighted sum of all patch features to aggregate the case features. Finally, a linear layer is utilized to predict the patient’s prognostic outcome as


$${𝐩}_{WSI}={𝐖}_{WSI}\cdot (\sum_{i=\text{0}}^{N}\;S_{i}\cdot {𝐟}_{i})$$

where ${𝐖}_{WSI}$ denotes trainable weight matrices.

Tissue regions with high attention scores from WSINet were segmented using SegNet, which consists of two sequential patch-level classification models: TNTM and TLSM. TNTM initially classifies tissue tiles into TLS, normal, and tumor categories, while TLSM further categorizes the TLS regions identified by TNTM into specific TLS phenotypes. Both models utilize ResNet18 as the backbone architecture for patch classification. The results from TNTM are concatenated to form a tissue prediction heatmap for each WSI. After training TLSM, its model parameters were fixed, and TLS morphological features were extracted for further analysis.

MVNet integrates the multi-view prognostic features extracted by SegNet. It consists of two branches: the Spatial Branch (SpB) and the Morphological Branch (MpB). SpB utilizes the TLS tissue distribution heatmap for each WSI, which is generated by SegNet, as input to the model. Specifically, this heatmap provides a spatial representation of TLS tissue blocks within the WSI. Features capturing the spatial characteristics of these TLS tissue blocks—including their positions on the heatmap, spatial relationships with adjacent tumor tissues, as well as their area and subtype information—are extracted. These comprehensive spatial features are then processed using a multi-layer perceptron, resulting in the spatial feature vector ${𝐫}_{S}$. The MpB employs TLSM to convert the TLS images obtained from SegNet into features ${𝐫}_{TLSM}$. These features are clustered for each individual case into 7 distinct categories. The cluster centers derived from this process serve as the representative lymphoid features for each patient [23]. Through clustering, the model’s training batch size is no longer restricted to 1. Increasing the batch size allows for more stable training of the model. These features are then processed using a simplified attention network, similar to the one employed in WSINet, to obtain the final features ${\hat{𝐫}}_{M}$ for each case. The visualization of the MpB architecture is illustrated in S3b Fig. The features from both branches are concatenated to form the multi-view feature representation ${𝐫}_{mv}\;$for each patient. A feature fusion prediction module further integrates these features, i.e.,


$${𝐟}_{fusion}=concatenate(({𝐖}_{fusion}\cdot ({𝐟}_{S}⊙{𝐟}_{mvS})),({𝐖}_{fusion}\cdot ({𝐟}_{M}⊙{𝐟}_{mvM})))$$

where ${𝐟}_{fusion}$ represents the deeply fused feature vector that integrates both spatial and morphological information. ${𝐟}_{S}$ and ${𝐟}_{M}$ are obtained by applying linear transformations to ${𝐫}_{S}$ and ${\hat{𝐫}}_{M}$ using different weight matrices. ${𝐟}_{mvS}$ and ${𝐟}_{mvM}$ are the multi-view features, obtained by applying two separate linear transformations to ${𝐫}_{mv}$, respectively. ${𝐖}_{fusion}$ is the weight matrix used for the final fusion of multi-modal features.

The two fused feature vectors are concatenated into a single vector, which is then used for further prediction. Finally, a linear layer is employed to perform prognostic classification. The architecture of MVNet is depicted in S3a Fig.

The final network, Multi-Modal Fusion (MMF), integrates clinical data through a four-layer multi-layer perceptron. Fourteen clinicopathological variables were concatenated into a 14-dimensional vector and passed through four fully connected (FC) layers, each followed by a BatchNorm1d layer and a ReLU activation function, resulting in a 32-dimensional embedding (S3a Fig). Notably, the network was not trained with a separate target; its parameters were optimized end-to-end along with the multimodal model using the final prediction loss. This clinical embedding was concatenated with the 32-dimensional output from the penultimate FC layer of MVNet to form a 64-dimensional fused feature vector, which was processed by a final FC layer to produce the MMF prediction score. Clinical features are concatenated with ${𝐟}_{fusion}$, and a linear layer is employed for the final prognostic prediction. By integrating multi-view TLS features and clinical data, SurvFinder provides precise prognostic predictions for patients with stage II CRC.

### Training and evaluation

During the model development phase, the prediction tasks in SurvFinder varied depending on the specific model. For WSINet and MVNet, predictive labels were based on relapse status, which included recurrence, metastasis, or death related to stage II CRC. In SegNet, the TNTM classifier was tasked with predicting tissue types (TLS, normal tissue, and tumor tissue), while the TLSM classifier focused on TLS subtypes (Agg, FL-1, FL-2). To ensure robust predictions, the Internal-CRCII dataset was split into five equal parts at the case level, and a 5-fold cross-validation strategy was applied. This consistent data division across the different models ensured the comparability of results. Notably, all models—including WSINet, SegNet, MVNet, and MMF—were exclusively developed and internally validated using the Internal-CRCII dataset. To avoid any form of information leakage or adaptation bias, the trained models were directly applied, without retraining or parameter adjustment, to three independent external validation cohorts. All prognostic models were trained for 500 epochs, while predictive models were trained for 100 epochs, with all model parameters trainable during the process [24]. The number of training epochs was determined empirically based on the performance and loss trends observed in the training and validation sets.

WSINet was initialized with random parameters and trained using the AdamW optimizer with weight decay. The cross-entropy loss function was used for optimization. The learning rate started at 0, followed by a linear warm-up for the first 10 epochs, peaking at 1e−3, and then decayed to 0 via a cosine annealing schedule by the end of training.

SegNet consists of two classifiers: TNTM (for tissue type classification) and TLSM (for TLS subtype classification). TNTM was trained on annotated tissue tiles (TLS, normal, and tumor) from WSI regions. Various augmentation techniques were applied, such as resizing, random rotations, vertical and horizontal flips, and color jittering (adjusting brightness, contrast, and saturation/hue). The model was initialized with pre-trained ResNet18 weights from ImageNet and optimized using the SGD optimizer with a momentum of 0.9, a weight decay of 1e−5, and a batch size of 128. The loss function used was cross-entropy. TLSM was trained on stitched TLS images (512 × 512 pixels) derived from TNTM’s prediction heatmaps. Similar to TNTM, TLSM was initialized with pre-trained ResNet18 weights and trained with the same setup.

MVNet comprises two branches: the MpB and the SpB. Both branches were initially trained with randomly initialized weights, while the entire MVNet model was trained with pre-trained weights from MpB and SpB. The Adam optimizer was used with an initial learning rate of 1e-3 and a cross-entropy loss function. Class weights were set at 0.5 for “No relapse” and 1.2 for “Relapse” to address class imbalance. An inverse time decay strategy (decay rate: 9e−3) was used to improve training stability and efficiency. Training was conducted with the full dataset in a single batch. The MMF model integrated clinical information with the MVNet features. The MMF training settings were kept consistent with MVNet for fair comparison, including epoch number, optimizer (Adam), loss function (cross-entropy), and class weights. A cosine learning rate decay with linear warm-up over 10 epochs and a batch size of 128 were employed. A batch size of 128 was used. For internal validation, each fold served as the test set once, with a model trained exclusively on the remaining folds. Predictions from all five folds were concatenated to form the complete internal test set. For external validation, the predicted probabilities from all five cross-validated models were first averaged to form an ensemble prediction, and only this ensemble prediction was binarized using the threshold to obtain the final categorical prediction. This ensemble approach was consistently applied to all external cohorts for both risk stratification and survival analyses. In this study, the stratification thresholds were calculated separately for each dataset. We further evaluated the stratification performance of the model under multiple fixed thresholds, and the results show that MVNet achieves highly significant stratification across all datasets, regardless of whether the thresholds are fixed or dataset-specific (S14 Fig and S3 Table). All patients with follow-up <24 months and no events were included in the survival analyses with their actual follow-up time and event status. All DL experiments were executed on four NVIDIA GeForce RTX 2080 Ti GPUs.

### Feature construction for MVNet

To synthesize the multi-view information of TLSs automatically, we extracted both morphological and spatial TLS features using the developed SegNet. Based on the patch-level probabilities from the multi-tissue classifier TNTM, a prediction heatmap was generated for each WSI, indicating the predicted regions of TLSs, normal tissue, and tumor tissue to visualize tissue characteristics. Considering that isolated tiny tissue tiles resulting from mispredictions could cause statistical deviations in distance and area information, a series of image post-processing was adopted to mitigate the effects of noise while maintaining the original characteristics of the tissue heatmaps. The post-processing included removing normal tissue domains from the heatmap, removing connected domains consisting of a single pixel and tumor tissue domains smaller than the threshold, and filling gaps within connected domains. The threshold was set to 20 pixels in this study, based on relevant guidelines and previous literature [25–27]. To demonstrate whether this processing strategy altered the spatial distribution of various components, we randomly selected 50 slides from each dataset and calculated the similarity of their heatmaps before and after processing using the structural similarity index measure (SSIM) method (S15 Fig). This analysis was performed after model evaluation and was independent of any model training or prediction processes. Statistical results showed that the average similarity of the heatmaps before and after processing in each dataset exceeded 0.92, indicating that our processing strategy effectively reduced statistical errors while preserving the original structural information of the heatmaps. The post-processed heatmaps were then utilized for feature construction. For each closed TLS region on the heatmap, the morphological feature was extracted using the pretrained TLS subtype classifier TLSM, along with the predicted phenotype status. Next, the spatial features were constructed, including area, quantity, phenotypes, and relative distances to tumor regions of TLS structures. We analyzed the frequency distribution of the number of TLSs within different ranges of TLS areas, distances of TLSs to the tumor edge, and TLS subtypes across all slides in the four datasets (S16 Fig). The results showed similar distributions of these three metrics across the four datasets, thereby eliminating potential biases due to data collection and indirectly demonstrating the consistent performance of SegNet across datasets from various sources. This analysis was not involved in any stage of model training or evaluation. The continuous variables of distance and area are converted into categorical variables to facilitate subsequent feature construction. Specifically, the distances from the centers of TLSs to the tumor margin were categorized into seven distinct, non-overlapping intervals: ≤0 mm, (0, 0.4] mm, (0.4, 1.0] mm, (1.0, 2.2] mm, (2.2, 4.5] mm, (4.5, 6.4] mm, and >6.4 mm. For External-CRCII-1, the number of TLSs located outside the tumor area was evenly distributed across each distance interval. A uniform interval setting was applied to each dataset. Similarly, the continuous values of TLS area were divided into seven distinct, non-overlapping intervals: (0, 0.03] mm^2^, (0.03, 0.05] mm^2^, (0.05, 0.07] mm^2^, (0.07, 0.10] mm^2^, (0.10, 0.15] mm^2^, (0.15, 0.28] mm^2^, and >0.28 mm^2^. According to the total area and quantity of the three TLS subtypes pertaining to each bin, a total of 226 features were designed to describe the spatial information. The number of segmentation bins was set to balance capturing spatial heterogeneity with maintaining computational stability, with reference to previous studies [28]. The detailed summary was shown in S9 Table.

### Interpretability

Both attention- and attribution-based interpretability methods were performed for the explanation of SurvFinder. The best performing model in the cross-validation was selected for observation. For WSINet, the attention scores obtained from the attention layers were normalized, signifying the relative importance of all inputted WSI tiles. The top 50 tiles with the highest attention scores were selected as representative patches for each patient. These highlighted tiles from correctly predicted ‘Relapse’ and ‘No relapse’ patients were aggregated and clustered into five clusters separately. The adopted clustering method was *k*-means, with *t*-SNE used for visualization. For each cluster, specific morphological characteristics were summarized by an experienced pathologist, resulting in the identification of 10 prognosis-related morphological features.

Due to the differing structures of the model branches, disparate interpretability methods were applied to the two branches of MVNet. The interpretation of the MpB was similar to that of WSINet, where the feature with the highest attention for each case was extracted for subsequent statistical analysis. The Shapley Additive Explanation-based method (SHAP) was employed for the SpB [29–31]. SHAP-styled attribution decision plots were used to visualize the attribution weights and direction of spatial features for further analyses.

### Statistical analysis

The study hypotheses and planned analyses were predefined. All planned analyses were conducted as described, and any deviations from the original plan, including exploratory or data-driven analyses, are clearly indicated. Statistical methods used are described below. SurvFinder was trained and validated using patient-level 5-fold cross-validation, ensuring consistent data splits across all models. Predictive model performance was evaluated using sensitivity, specificity, and the area under the receiver operating characteristic curve (AUROC). Prognostic models were assessed using the AUROC. High-risk and low-risk groups, stratified by prognostic models, were based on the predicted class (1 for high-risk and 0 for low-risk). The cutoff threshold of the DL model’s ROC curve was determined by the Equal Error Rate criterion to dichotomize the model’s probabilities into binary predictions. The primary endpoint of this study was relapse-free survival (RFS), defined as the time from the date of surgery to the date of first disease recurrence, metastasis or death from tumor. Statistical tests included chi-squared tests for confusion matrices, DeLong’s test for AUC comparisons, and log-rank tests for survival curves. Univariable and multivariable Cox regression analyses were used to identify prognostic factors, with significance defined as *p* < 0.05. Box plots displayed the 25th, 50th, and 75th quantiles. All statistical analyses were conducted in R version 4.2.1 (2022-06-23). Deep learning models were implemented in Python 3.8.12 using the PyTorch 1.10.1 framework. This study is reported as per the Strengthening the Reporting of Observational Studies in Epidemiology (STROBE) guideline (S1 Checklist) and the Transparent Reporting of a multivariable prediction model for Individual Prognosis Or Diagnosis (TRIPOD) guideline (S2 Checklist).

## Results

### Study design and patient cohorts

The SurvFinder framework, as outlined in Fig 1, was developed and validated using data from four distinct patient cohorts. Following rigorous data filtering, we included a total of 6,950 H&E-stained pathology slides from 1,604 patients for model development. The detailed inclusion and exclusion criteria are provided in the Methods section. The Internal-CRCII cohort, which was used for model training and internal validation, comprised 3,494 slides from 743 stage II CRC patients from a single medical center. For external validation, we utilized the External-CRCII-1 and External-CRCII-2 cohorts, which consisted of 1,315 slides from 352 cases and 1,708 slides from 331 cases, respectively, collected from two independent medical centers. To ensure accurate representation of the tumor microenvironment, each case in both internal and external datasets included at least three formalin-fixed paraffin-embedded slides containing tumor tissue. Additionally, the TCGA-CRCII cohort, derived from the TCGA-COAD and TCGA-READ projects, included 433 slides from 178 stage II cases. A comprehensive description of these datasets is provided in S1 Fig.

### WSINet identifies prognosis-related tissue biomarkers from WSIs

**Fig 2:**
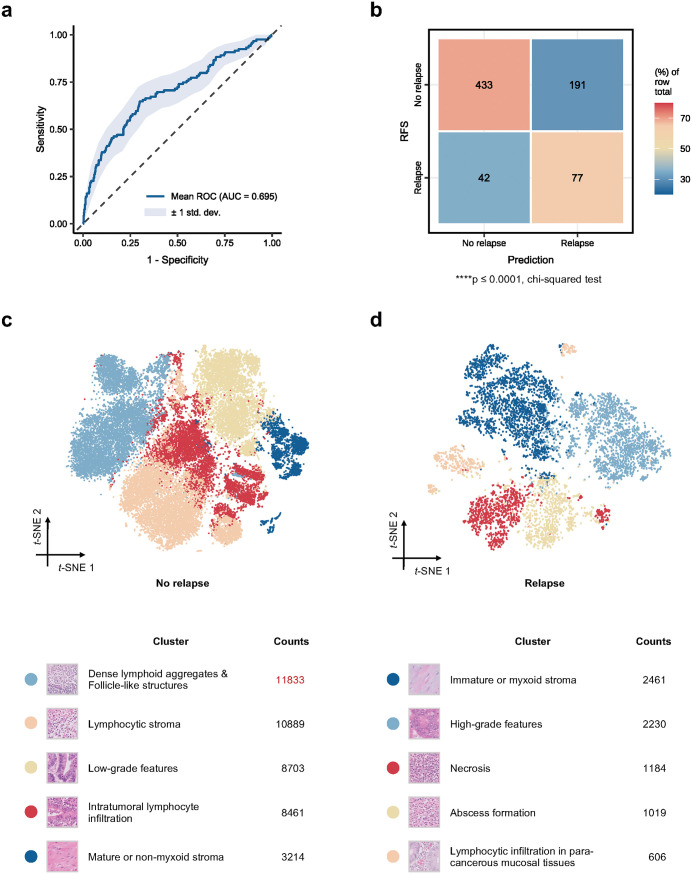
The performance and interpretation of WSINet for identifying prognosis-associated tissue biomarkers. **(a)** Precision–recall and receiver operating characteristic (ROC) curves of WSINet, illustrating the mean performance across a 5-fold cross-validation. **(b)** Confusion matrix detailing the predictions. **(c, d)** Clustering results of highlighted tissue biomarkers for the ‘no relapse’ group (c) and ‘relapse’ group (d) using the *k*-means clustering method. This includes *t*-SNE plots (top), cluster characteristic descriptions, and patch counts for each cluster (bottom). The chi-squared test was used to measure predictive performance. Statistical significance is indicated as follows: ns, *p* > 0.05; *p ≤ 0.05; **p ≤ 0.01; ***p ≤ 0.001; ****p ≤ 0.0001. ns, not significant; RFS, relapse-free survival.

To identify tissue features relevant to prognosis from WSIs, we developed WSINet, an attention-based multiple-instance learning model that serves as the initial component of the SurvFinder framework. WSINet aggregates features from all WSIs of each case to generate a patient-level input for prognosis prediction. The model was trained and evaluated on the Internal-CRCII dataset using 5-fold cross-validation, achieving an AUROC of 0.695 (95% confidence interval [CI] [0.639,0.751]), indicating a statistically significant correlation between model predictions and actual relapse status (Fig 2a and 2b). The attention mechanism in WSINet was leveraged to identify high-attention tissue regions, which were then analyzed using *k*-means clustering to delineate five significant tissue biomarkers for both no-relapse and relapse groups (Fig 2c and 2d). Notably, the largest cluster within the no-relapse group was enriched for regions exhibiting dense lymphoid aggregates and follicle-like architecture, morphological hallmarks of TLSs (S2 Fig). Importantly, all clustering and feature identification analyses were restricted to the internal development cohort. The external datasets were used exclusively for independent validation, ensuring no data leakage and preserving the integrity of the evaluation process. Taken together, these results suggest that TLSs could serve as key favorable prognostic tissue biomarkers in stage II CRC.

### SegNet automatically segments multi-class tissue biomarkers

**Fig 3:**
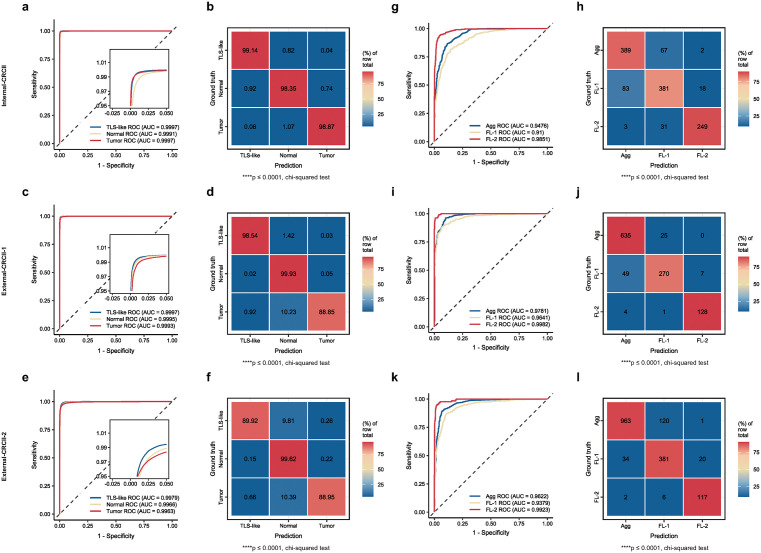
Segmentation performance of SegNet for multi-class tissue biomarkers. **e** ROC curve of TNTM, the first component of SegNet, for segmenting TLSs, normal, and tumor tissue on Internal-CRCII. **(b)** Confusion matrix of TNTM predictions on Internal-CRCII, where each cell represents the percentage of the total count of the true labels. **(c, d)** ROC curve (c) and confusion matrix (d) of TNTM on External-CRCII-1. **(e, f)** ROC curve (e) and confusion matrix (f) of TNTM on External-CRCII-2. **(g)** ROC curve of TLSM, the second component of SegNet, for identifying TLS phenotypes on Internal-CRCII. **(h)** Confusion matrix of TLSM performance on Internal-CRCII. **(i, j)** ROC curve (i) and confusion matrix (j) of TLSM on External-CRCII-1. **(k, l)** ROC curve (k) and confusion matrix (l) of TLSM on External-CRCII-2. The chi-squared test was used to measure predictive performance. Statistical significance is indicated as follows: ns, *p* > 0.05; *p ≤ 0.05; **p ≤ 0.01; ***p ≤ 0.001; ****p ≤ 0.0001. ns, not significant; TLS, tertiary lymphoid structure; TNTM, TLS-normal-tumor segmentation model; TLSM, TLS subtyping model; SegNet, segmentation network; Agg, aggregates; FL-1, primary follicles; FL-2, secondary follicles; Internal-CRCII, internal colorectal cancer stage II cohort; External-CRCII-1, external colorectal cancer stage II cohort 1; External-CRCII-2, external colorectal cancer stage II cohort 2.

To further categorize and segment TLS regions identified by WSINet, as well as other tissue biomarkers such as tumor tissue, we developed SegNet, a patch-level multi-tissue classification network. SegNet comprises two models: the TLS-normal-tumor tissue classification (TNTM) and the TLS phenotype classification (TLSM). Both models were trained on Internal-CRCII and validated on both internal and external datasets. The TNTM model demonstrated excellent performance with AUROCs of 0.9997 (95% CI [0.9997,0.9997]) for TLS-like tissue, 0.9991 (95% CI [0.9991,0.9992]) for normal tissue, and 0.9997 (95% CI [0.9996,0.9997]) for tumor tissue on the Internal-CRCII dataset (Fig 3a and 3b), with similarly high performance on External-CRCII-1 and External-CRCII-2, confirming the model’s robustness across different datasets (Fig 3c–3f). Detailed performance metrics are provided in S1 Table.

The TLSM model was designed to further classify TLSs into three distinct phenotypes: aggregates (Agg), primary follicles (FL-1) and secondary follicles (FL-2) [32]. On Internal-CRCII dataset, the TLSM model achieved AUROCs of 0.9476 (95% CI [0.9366,0.9586]) for Agg, 0.91 (95% CI [0.8941,0.9259]) for FL-1, and 0.9851 (95% CI [0.9787,0.9916]) for FL-2. Similar performance was observed in the External-CRCII-1 and External-CRCII-2 datasets (Fig 3g–3l and S2 Table). These results validate SegNet’s ability to accurately segment and subtype tissue biomarkers, laying a histological foundation for further extraction and integration of multi-view prognostic features from WSIs.

### MVNet predicts stage II CRC patients’ prognosis via multi-view combination

**Fig 4:**
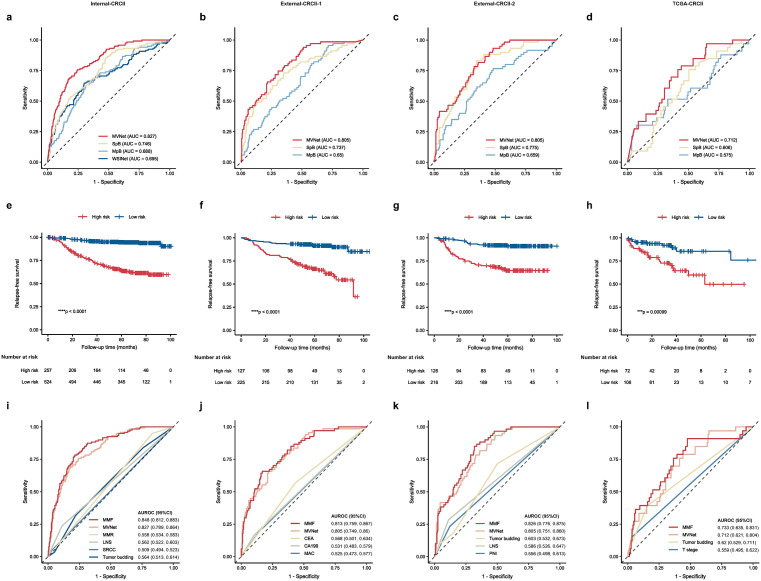
The predictive and prognostic performance of SurvFinder. **(a)** ROC curves of WSINet, MpB, SpB, and MVNet on Internal-CRCII. **(b–d)** ROC curves of the MpB, SpB, and MVNet on External-CRCII-1 (b), External-CRCII-2 (c), and TCGA-CRCII (d), respectively. **(e–h)** Kaplan–Meier (K–M) survival curves for all cases stratified by the predicted risk from MVNet, on Internal-CRCII (e), External-CRCII-1 (f), External-CRCII-2 (g), and TCGA-CRCII (h), respectively. Censors are indicated with a ‘+’. **(i–l)** ROC curves comparing the prognostic capacity of MMF with MVNet and other significant clinicopathological factors on Internal-CRCII (i), External-CRCII-1 (j), External-CRCII-2 (k), and TCGA-CRCII (l), respectively. The log-rank test was used to calculate statistical significance. Statistical significance is indicated as follows: ns, *p* > 0.05; *p ≤ 0.05; **p ≤ 0.01; ***p ≤ 0.001; ****p ≤ 0.0001. ns, not significant; SpB, spatial feature branch; MpB, morphological feature branch; MVNet, multi-view network; MMF, multi-modal fusion; MMR, mismatch repair; LNS, lymph node sampling; SRCC, signet-ring cell carcinoma; MAC, mucinous adenocarcinoma; PNI, perineural invasion; Internal-CRCII, internal colorectal cancer stage II cohort; External-CRCII-1, external colorectal cancer stage II cohort 1; External-CRCII-2, external colorectal cancer stage II cohort 2; TCGA-CRCII, TCGA colorectal cancer stage II cohort.

We then developed MVNet, a multi-view fusion model that integrates spatial and morphological characteristics of TLSs for prognosis prediction. MVNet consisted of three components: a SpB, a MpB, and a fusion predictor (S3 Fig). The predictive performance of these models was compared across all datasets (Fig 4a–4d). The MpB, trained on latent features derived from TLSM, achieved AUROCs of 0.688 (95% CI [0.637,0.739]), 0.65 (95% CI [0.581,0.719]), and 0.659 (95% CI [0.584,0.734]) on the Internal-CRCII, External-CRCII-1, and External-CRCII-2 datasets, respectively. The SpB, trained on spatial features such as distance, area, and phenotypes of TLS, achieved AUROCs of 0.746 (95% CI [0.699,0.793]), 0.737 (95% CI [0.667,0.808]), and 0.775 (95% CI [0.717,0.833]) on the same cohorts. Combining spatial and morphological features in the fusion predictor resulted in AUROCs of 0.827 (95% CI [0.789,0.864]), 0.805 (95% CI [0.749,0.860]), and 0.805 (95% CI [0.748,0.861]), demonstrating a statistically significant concordance between the MVNet predictions and the ground truth (S4 Fig). MVNet consistently outperformed both WSINet and the single-branch models, proving the effectiveness of multi-view integration for prognostic assessment (S5 Fig).

### MVNet surpasses and complements clinicopathological prognostic factors in stage II CRC

To assess MVNet performance in a real-life scenario, we first evaluated the RFS in the high- and low-risk subgroups predicted by MVNet using Kaplan–Meier (K–M) analysis. Significant survival differences (*P* < 0.0001) were observed across all datasets in log-rank tests (Fig 4e–4h). Next, we compared MVNet with 16 clinicopathological indicators frequently used for risk stratification as well as ACT status in stage II CRC. In univariate Cox regression analysis on Internal-CRCII, four factors were significant, including tumor budding (*P* = 0.044), MMR (*P* = 0.003), lymph node sampling (LNS, *P* < 0.001) and MVNet (*P* < 0.001) (S6 Fig). LNS was defined as the number of lymph nodes examined during surgery, with <12 nodes considered insufficient, in accordance with NCCN guidelines [3,4]. In multivariate Cox regression, MVNet demonstrated the highest hazard ratio (HR: 8.23, 95% CI: 5.43–12.47; *p* < 0.001), outperforming other significant factors (S6 Fig). These results confirmed that MVNet is a strong independent prognostic factor, significantly outperforming other established biomarkers. The correlation analysis also validated the independence of the model’s prediction (S7 Fig). Similar findings were observed in external datasets (S6c–S6h and S7b–S7d Figs). Additionally, the model’s performance was evaluated across multiple fixed thresholds (S3 Table).

MVNet consistently showed superior prognostic performance, establishing itself as an independent and robust predictor. To explore the potential for combining MVNet with clinicopathological data, we developed a MMF model. This fusion model exhibited consistently higher AUROCs than both MVNet and the clinical-only model across all datasets (Figs 4i–4l and S8), confirming the complementary nature of SurvFinder with established biomarkers.

Additionally, we integrated MVNet into nomograms alongside clinicopathological factors (S7e–S7h Fig), with calibration curves closely aligning with ideal predictive models (S7i–S7l Fig). These findings indicate that MVNet enhances the predictive power of established clinicopathological biomarkers, confirming its potential as both an independent prognostic tool and a complementary asset in the clinical management of in stage II CRC.

### MVNet’s potential to inform ACT decisions in stage II CRC

**Fig 5:**
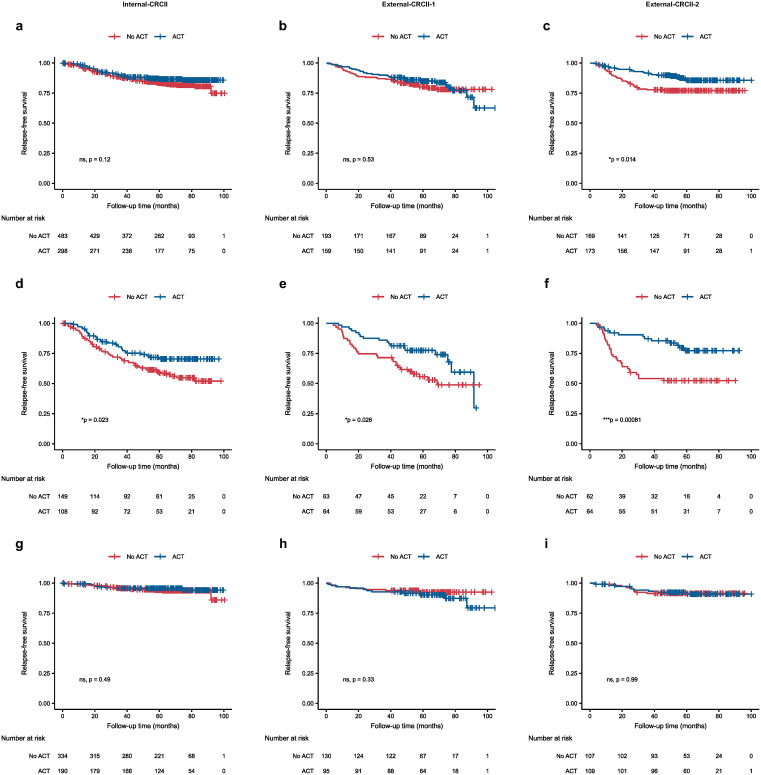
Performance of SurvFinder for decision-making of adjuvant chemotherapy. **(a–c)** Kaplan–Meier (K–M) curves for all patients from Internal-CRCII (a), External-CRCII-1 (b), and External-CRCII-2 (c), stratified by the receipt status of adjuvant chemotherapy (ACT). Censors are indicated with a ‘+’. **(d–f)** Kaplan–Meier (K–M) survival curves for cases predicted as high risk by MVNet, stratified by the receipt status of ACT, on Internal-CRCII (d), External-CRCII-1 (e), and External-CRCII-2 (f), respectively. The log-rank test was used to calculate statistical significance. Statistical significance is indicated as follows: ns, *p* > 0.05; *p ≤ 0.05; **p ≤ 0.01; ***p ≤ 0.001; ****p ≤ 0.0001. ns, not significant; ACT, adjuvant chemotherapy; Internal-CRCII, internal colorectal cancer stage II cohort; External-CRCII-1, external colorectal cancer stage II cohort 1; External-CRCII-2, external colorectal cancer stage II cohort 2.

We next explore MVNet’s potential to inform ACT decisions in stage II CRC. First, we assessed the impact of ACT on prognosis across different datasets (Fig 5a–5c). Although a significant association between ACT and improved prognosis was observed in the External-CRCII-2 cohort (*p* = 0.014), K–M analysis indicated no significant improvement in prognosis with ACT in the Internal-CRCII (*p* = 0.12) and External-CRCII-1 datasets (*p* = 0.53). Subsequently, we analyzed the MVNet-predicted high-risk subgroup (Fig 5d–5f). In this group, ACT was significantly associated with a favorable prognostic response in Internal-CRCII (*p* = 0.023) and External-CRCII-1 (*p* = 0.026) cohorts. Notably, in the External-CRCII-2 cohort, the high-risk group showed even stronger statistical significance and greater benefit in the K–M curve (*p* = 0.00081) compared to the full cohort. In contrast, MVNet-predicted low-risk patients derived no significant survival benefit from ACT in any cohort (Fig 5g–5i). In addition, MVNet consistently served as a significant prognostic factor across both the ACT and no-ACT subgroups (S4 Table and S9 Fig). The distribution of clinicopathological high-risk features across each subgroup is presented in S5 Table. We further performed Cox proportional hazards models including an interaction term between ACT and MVNet risk group. Although no significant interaction was observed in the internal dataset, significant or near-significant interactions were identified in two external cohorts (S6 Table). These results suggest that MVNet may serve as a clinically meaningful tool for guiding ACT decisions in stage II CRC.

### XAI on SurvFinder unveils prognostically relevant TLS characteristics

**Fig 6:**
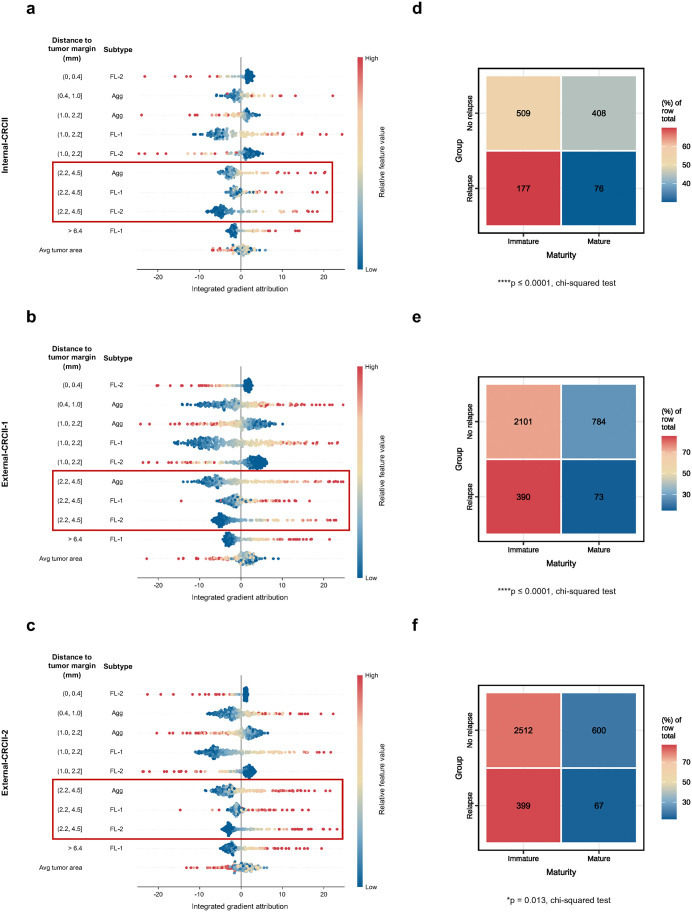
Interpretation of SurvFinder. **(a–c)** Top 10 global spatial feature attributions to all predictions, sorted by distances to the tumor margin and subtypes for Internal-CRCII (a), External-CRCII-1 (b), and External-CRCII-2 (c), respectively. Each attribution feature represents the exact contribution of a TLS phenotype distributed within specific distances to the invasive margin of the tumor. **(d–f)** Confusion matrices on Internal-CRCII (d), External-CRCII-1 (e), and External-CRCII-2 (f), comparing the maturity differences of high-priority TLS morphological features extracted from ‘relapse’ and ‘no relapse’ groups. The chi-squared test was used to measure differences. Statistical significance is indicated as follows: ns, *p* > 0.05; *p ≤ 0.05; **p ≤ 0.01; ***p ≤ 0.001; ****p ≤ 0.0001. ns, not significant; Internal-CRCII, internal colorectal cancer stage II cohort; External-CRCII-1, external colorectal cancer stage II cohort 1; External-CRCII-2, external colorectal cancer stage II cohort 2.

To enhance the interpretability of SurvFinder and better understand the prognostically relevant features learned by the model, we applied XAI techniques to the risk prediction models, as detailed in the Methods section. The top 10 spatial features contributing to prognosis were identified across internal and external datasets (Fig 6a–6c). Most of these spatial characteristics were associated with favorable prognosis, whereas the mean tumor area per slide correlated with poorer prognosis. These findings align with existing research in CRC and confirm that SurvFinder is grounded in established biological principles [33–35]. Interestingly, more than half of the identified features were located between 1 and 4.5 mm from the tumor margin, suggesting that TLSs in this region are critical for prognosis. Furthermore, TLS subtypes located 2.2–4.5 mm from the tumor margin consistently correlated with favorable prognosis, indicating that TLSs in this area may serve as important indicators of low-risk outcomes in stage II CRC.

We also investigated the morphological features of TLSs highlighted by the model. The attention maps revealed that mature TLSs (FL-2) were more prevalent in the non-relapse group, while immature TLSs (Agg and FL-1) were more frequent in the relapse group (Fig 6d–6f). This pattern was consistent across all datasets, suggesting that TLS maturity is positively correlated with better prognosis in stage II CRC. The WSI visualizations of the interpretable results are presented in S10 Fig. Overall, the interpretation analyses support the biological relevance of the features used by SurvFinder and provide new insights into the role of TLSs in stage II CRC prognosis.

### SurvFinder’s predictive accuracy in TCGA dataset

To validate the generalizability of SurvFinder, we tested its predictive and prognostic performance on the TCGA dataset. Prognostic predictions in the TCGA-CRCII cohort yielded AUROCs of 0.575 (95% CI [0.455,0.694]) for the MpB, 0.606 (95% CI [0.508,0.703]) for the SpB, and 0.712 (95% CI [0.621,0.804]) for the MVNet (Fig 4d). These results demonstrate that SurvFinder maintains robust performance in the TCGA dataset (S4d and S5d Figs), with stable risk stratification as shown in the K–M curves (Fig 4h).

Due to incomplete clinical data in the TCGA cohort, Cox regression analyses were performed using only available clinicopathological factors and the MVNet score. In the univariate Cox analysis, five factors were statistically significant: tumor budding (*p* = 0.007), Grading (*p* = 0.037), T stage (*p* = 0.002), BD3 (*p* = 0.034) and MVNet (*p* = 0.002) (S6g Fig). Multivariate Cox regression confirmed MVNet as an independent prognostic factor (S6h Fig). MVNet continued to outperform other clinicopathological parameters and maintained stable performance in multimodal data integration (Figs 4l, S7d, S7h, and S7l). These results underscore SurvFinder’s ability to stratify risk and predict outcomes across diverse patient populations, supporting its potential generalizability across multiple datasets.

## Discussion

H&E-stained slides present an invaluable opportunity for developing clinically applicable markers that can be widely used in risk stratification and therapeutic decision-making. In this study, we developed an interpretable DL framework to autonomously identify multi-view histological marker features from WSIs. The DL-derived markers demonstrated consistent performance across four multicenter datasets, surpassing several conventional clinicopathological parameters in predictive accuracy and providing complementary biological insights. A key finding was the model-driven identification of tumor-associated TLSs as favorable prognostic indicators. This highlights the potential of AI-assisted pathology not only for automating feature extraction but also for uncovering biologically and clinically relevant tissue structures that may be under-recognized in routine manual assessment.

Our approach integrates DL with interpretability, providing clinicians intuitive and effective markers for clinical risk assessment and treatment response prediction. Despite the emergence of various genetic and molecular methods to aid in managing stage II CRC, their clinical application has remained limited [12,36], possibly due to their complexity, rarity of the condition, or outdated computational methods [24,37]. In contrast, our CPATH marker has the potential to streamline clinical decision-making by reducing time and costs, while showing consistent predictive performance across multicenter datasets. Depending on data heterogeneity, both adaptive and unified thresholding strategies may be viable approaches for aligning model predictions with clinical risk assessment frameworks. Although DL-based biomarker assessment introduces additional computational costs, it enables standardized, high-throughput evaluation with reduced labor and inter-observer variability. While CPATH shows promise in predicting adjuvant therapy needs, its advantage over other genetic biomarkers such as KRAS and BRAF requires further validation [38,39]. The stratification analysis for ACT response is exploratory and requires further validation in prospective studies.

Several previous studies have attempted to develop CPATH models for CRC prognosis, though many have struggled with stage II CRC specifically. For example, Foersch, S. and colleagues developed a multistain DL model to predict CRC prognosis across all stages but found limited success within stage II patients [24]. Similarly, Kleppe, A. and colleagues created a DL-based clinical decision support system [40], but the lack of interpretability in their model hindered its clinical adoption [41,42]. Our model addresses this gap by highlighting high-priority tissue regions, thereby enhancing clinicians’ understanding of the model’s reliability and facilitating the identification of additional histological markers, such as TLS in this study. Despite the model’s complexity, embedded interpretability components support clinical insight, and future work may integrate these with simplified decision frameworks to aid deployment. While earlier models explored tumor–adipose interactions with interpretable methods, their validation was limited to single-center datasets [43,44]. Our study extends this by performing multicenter external validation across four independent cohorts and exploring potential treatment relevance.

The main contribution of this study lies in revealing the potential association between TLS and prognosis as well as chemotherapy benefit in stage II CRC through an automated analysis pipeline. Given that not all data are IHC-supported, we refer to these regions as “TLS-like” throughout this study. TLSs, known to enhance immune cell infiltration into the tumor microenvironment, have demonstrated promising prognostic value in various cancer types [45,46]. Their association with ACT benefit and prognosis in stage II CRC remains to be further explored. Additionally, while previous models used single parameter [47], our model demonstrates that a comprehensive view, encompassing both spatial distribution and maturity, provides deeper prognostic insights. In our findings, spatial features, particularly the distribution of TLSs, outperformed morphological characteristics in predicting prognosis for stage II CRC. Our current study suggests that TLSs located 1–4.5 mm from the tumor margin are critical indicators of favorable outcomes. The role of TLS maturity in prognosis was also highlighted, aligning with previous studies linking TLS maturity to reduced recurrence risk in early-stage CRC [48]. The interaction between TLS cellular composition and spatial distribution warrants further exploration, particularly in relation to its effect on patient outcomes. Future investigations could systematically evaluate additional histological structures revealed by the model, which may complement TLS-based features and further strengthen clinical utility.

Our study also provides an effective approach for weakly supervised medical analysis, effectively capturing critical tissue features in WSIs. Weakly supervised learning is common in medical imaging but often faces challenges [49], such as the introduction of noise when whole-slide labels are applied to image patches [21,50,51]. Although direct prediction of patient outcomes from image patches is theoretically possible, it remains challenging due to label noise and poor interpretability at the slide level; therefore, feature-based aggregation offers a more robust and interpretable solution [52]. While multiple instance learning (MIL) models address some of these limitations by focusing on key morphological features, they often overlook spatial relationships between tissue regions [21]. Our framework improves upon this by integrating both spatial and morphological characteristics of histological markers. Validation across multiple independent cohorts revealed that the spatial distribution of markers provides essential predictive insights, and the integration of multiple views enhances overall performance. The comparable success of the morphology-based and MIL models further indicates that our approach preserves predictive information without sacrificing robustness. The performance difference between MVNet and MIL-based WSINet may be attributed to the lack of spatial awareness in WSINet, its susceptibility to irrelevant tissue interference, and the underrepresentation of small yet informative structures such as TLSs in WSIs. Incorporating compensation mechanisms for low-abundance signals may further enhance the performance of WSINet [53,54]. These findings support the effectiveness of explicitly extracting and modeling key tissue features to enhance prognostic prediction.

Despite promising results, our study has limitations. As a retrospective multicenter analysis, it is subject to selection bias and confounding, limiting causal inference. Validation on prospective and publicly available datasets is warranted, and randomized trials remain the gold standard to establish clinical utility. Additionally, distributional shifts across cohorts highlight the need for future methods to enhance model generalizability, and the need for dataset-specific thresholds likewise limits applicability to unseen cohorts. Furthermore, our framework’s flexibility allows for further optimization depending on specific tasks. For instance, the incorporation of transformer architectures or graph convolutional network could enhance the model’s ability to learn holistic regional features [55,56]. Moreover, SurvFinder is fully automated for TLS segmentation and classification; however, for additional biomarkers not included in the current training, manual annotations are still required, limiting true end-to-end automation. Incorporating unsupervised learning could improve scalability and clinical applicability. Additionally, incorporating more detailed chemotherapy regimen information, along with multidimensional data such as cellular composition and genomic profiles, may further enhance the model’s predictive performance.

In addition to these methodological considerations, important aspects regarding robustness and clinical translation remain. Although the model performed consistently across the included cohorts, its stability under real-world variability—such as differences in scanner hardware, and tissue preparation—was not systematically evaluated. Furthermore, the framework has not yet been integrated into routine pathology workflows, limiting immediate applicability for clinical decision-making. Future work should include standardized quality-control pipelines, robustness stress-testing, and prospective deployment or decision-impact studies to confirm reliability and clinical utility.

In summary, we developed a DL model that autonomously identifies key histological markers for risk stratification and treatment prediction in stage II CRC. This approach facilitates pathologists in identifying critical tissue indicators and provides a scalable framework with potential to support AI-assisted clinical decision-making.

## Supporting information

S1 Fig CONSORT diagrams and baselines of study participants. CONSORT diagrams for data filtering and baseline characteristics of study participants in Internal-CRCII **(a)**, External-CRCII-1 **(b)**, External-CRCII-2 **(c)**, and TCGA-CRCII **(d)**, respectively. CRC, colorectal cancer; WSI, whole slide image; RFS, relapse-free survival; MMR, mismatch repair; Internal-CRCII, internal colorectal cancer stage II cohort; External-CRCII-1, external colorectal cancer stage II cohort 1; External-CRCII-2, external colorectal cancer stage II cohort 2; TCGA-CRCII, TCGA colorectal cancer stage II cohort.
(DOCX)

S2 Fig Representative patches from the most abundant cluster in the no-relapse group. Representative image patches from the cluster with the highest patch count in the no-relapse group. These patches predominantly display dense lymphoid aggregates and follicle-like structures, consistent with the morphology of tertiary lymphoid structures (TLSs). Each patch corresponds to a tissue area of 128 μm × 128 μm.
(DOCX)

S3 Fig Overview of MVNet, MMF and MpB branch. **(a)** Model architecture of MVNet and MMF, illustrating the integration of SpB, MpB and clinical parameters for multi-view analysis. **(b)** Model architecture of the MpB branch, focusing on morphological feature extraction within the MVNet framework. Fc(x) represents a fully connected layer with an output dimension x. WSI, whole slide image; MVNet, multi-view network; TLS, tertiary lymphoid structure; ACT, adjuvant chemotherapy; MpB, morphological feature branch; SpB, spatial feature branch; TLSM, TLS subtyping model; MMF, multi-modal fusion.
(DOCX)

S4 Fig Confusion matrices of MVNet predictions. Confusion matrices illustrating the performance of MVNet predictions for Internal-CRCII **(a)**, External-CRCII-1 **(b)**, External-CRCII-2 **(c)**, and TCGA-CRCII **(d)** cohorts. The chi-squared test was employed to assess performance. Statistical significance is denoted as follows: ns, *p* > 0.05; *p ≤ 0.05; **p ≤ 0.01; ***p ≤ 0.001; ****p ≤ 0.0001. ns, not significant; Internal-CRCII, internal colorectal cancer stage II cohort; External-CRCII-1, external colorectal cancer stage II cohort 1; External-CRCII-2, external colorectal cancer stage II cohort 2; TCGA-CRCII, TCGA colorectal cancer stage II cohort.
(DOCX)

S5 Fig Comparison of MVNet predictive performance. **(a)** Comparison of AUROCs among WSINet, two branches, and MVNet on the Internal-CRCII cohort. **(b–d)** Comparison of AUROCs among the two branches and MVNet on the External-CRCII-1 (b), External-CRCII-2 (c), and TCGA-CRCII (d) cohorts, respectively. DeLong’s test was used for statistical assessment. Significance is denoted as follows: ns, **p** > 0.05; *p ≤ 0.05; **p ≤ 0.01; ***p ≤ 0.001; ****p ≤ 0.0001. ns, not significant; SpB, spatial feature branch; MpB, morphological feature branch; MVNet, multi-view network; Internal-CRCII, internal colorectal cancer stage II cohort; External-CRCII-1, external colorectal cancer stage II cohort 1; External-CRCII-2, external colorectal cancer stage II cohort 2; TCGA-CRCII, TCGA colorectal cancer stage II cohort.
(DOCX)

S6 Fig Cox regression analysis of MVNet and clinicopathological parameters. **(a)** Univariate Cox regression analysis for Internal-CRCII. **(b)** Multivariate Cox regression analysis for Internal-CRCII. **(c, d)** Univariate (c) and Multivariate (d) Cox regression analyses for External-CRCII-1. **(e, f)** Univariate (e) and Multivariate (f) Cox regression analyses for External-CRCII-2. **(g, h)** Univariate (g) and Multivariate (h) Cox regression analyses for TCGA-CRCII. Statistical significance was calculated using the Wald test. MVNet, multi-view network; PNI, perineural invasion; VI, vascular invasion; SRCC, signet-ring cell carcinoma; MAC, mucinous adenocarcinoma; LNS, lymph node sampling; MMR, mismatch repair; ACT, adjuvant chemotherapy; BD3, tumor budding grade 1–3; Internal-CRCII, internal colorectal cancer stage II cohort; External-CRCII-1, external colorectal cancer stage II cohort 1; External-CRCII-2, external colorectal cancer stage II cohort 2.
(DOCX)

S7 Fig Correlation maps and nomograms of MVNet and clinicopathological parameters. **(a–d)** Correlation maps illustrating statistically significant clinical parameters and MVNet score for Internal-CRCII (a), External-CRCII-1 (b), External-CRCII-2 (c), and TCGA-CRCII (d), respectively. **(e–h)** Nomograms predicting the proportion of cases with relapse-free survival post-operation for Internal-CRCII (e), External-CRCII-1 (f), External-CRCII-2 (g), and TCGA-CRCII (h), respectively. **(i–l)** Calibration plots of the MVNet-clinic linear model comparing observed and predicted 5-year outcomes for Internal-CRCII (i), External-CRCII-1 (j), External-CRCII-2 (k), and TCGA-CRCII (l), respectively. Perfect performance is represented by points along the 45-degree line. MVNet, multi-view network; PNI, perineural invasion; SRCC, signet-ring cell carcinoma; MAC, mucinous adenocarcinoma; LNS, lymph node sampling; MMR, mismatch repair; RFS, relapse-free survival; Internal-CRCII, internal colorectal cancer stage II cohort; External-CRCII-1, external colorectal cancer stage II cohort 1; External-CRCII-2, external colorectal cancer stage II cohort 2; TCGA-CRCII, TCGA colorectal cancer stage II cohort.
(DOCX)

S8 Fig Predictive performance of clinicopathological feature–only model across datasets. **(a–c)** Receiver operating characteristic (ROC) curves of the clinicopathological feature–only model in Internal-CRCII (a), External-CRCII-1 (b), and External-CRCII-2 (c) cohorts, respectively. Internal-CRCII, internal colorectal cancer stage II cohort; External-CRCII-1, external colorectal cancer stage II cohort 1; External-CRCII-2, external colorectal cancer stage II cohort 2.
(DOCX)

S9 Fig Prognostic performance of SurvFinder stratified by adjuvant chemotherapy receipt. **(a–c)** Kaplan–Meier (K–M) survival curves for patients who received adjuvant chemotherapy (ACT+, ACT+ subgroup) from Internal-CRCII (a), External-CRCII-1 (b), and External-CRCII-2 (c), stratified by the predicted risk from MVNet. **(d–f)** Kaplan–Meier (K–M) survival curves for patients who did not receive adjuvant chemotherapy (ACT−, ACT− subgroup) from Internal-CRCII (d), External-CRCII-1 (e), and External-CRCII-2 (f), stratified by the predicted risk from MVNet. Censors are indicated with a ‘+’. The log-rank test was used to calculate statistical significance. Statistical significance is indicated as follows: ns, *p* > 0.05; *p ≤ 0.05; **p ≤ 0.01; ***p ≤ 0.001; ****p ≤ 0.0001. ns, not significant; ACT, adjuvant chemotherapy; Internal-CRCII, internal colorectal cancer stage II cohort; External-CRCII-1, external colorectal cancer stage II cohort 1; External-CRCII-2, external colorectal cancer stage II cohort 2.
(DOCX)

S10 Fig SurvFinder interpretation on WSIs from “No relapse” and “Relapse” Groups. **(a, b)** Typical WSI examples from the ‘No relapse’ group, showing SurvFinder interpretation results. Each includes the original H&E-stained WSI image (left), the WSI image overlaid with the SegNet-predicted heatmap (middle), and descriptions of the typical TLSs (right). **(c, d)** Typical WSI examples from the ‘Relapse’ group, following the same format. Each group includes two cases correctly predicted as low-risk and high-risk by SurvFinder, respectively. The scale bar in each image represents a length of 5 mm. TLS, tertiary lymphoid structure; SegNet, segmentation network.
(DOCX)

S11 Fig Classifications of TLS determined by immunohistochemical staining. Representative images of TLS classifications (Agg, FL-1, and FL-2) and immunohistochemical patterns stained with CD20, CD21, and CD10. The scale bar in each image represents 250 µm. Agg, aggregates; FL-1, primary follicles; FL-2, secondary follicles.
(DOCX)

S12 Fig Visualization of white background removal using Otsu’s method. Comparative visualization of tissue regions before and after background removal. **(a)** and **(c)** show tile regions after applying Otsu’s method, effectively excluding non-tissue white areas. **(b)** and **(d)** display the corresponding original whole-slide image thumbnails prior to processing.
(DOCX)

S13 Fig Comparison of predictive performance between stain-normalized and unstained images in External-CRCII-2. Predictive performance comparison on the External-CRCII-2 dataset. **(a)** AUROC curve based on stain-normalized images processed using the Vahadane method. **(b)** AUROC curve based on the corresponding unstained (original) images. The stained group consistently exhibited higher predictive accuracy than the unstained group.
(DOCX)

S14 Fig Prognostic performance of SurvFinder using an Internal-CRCII-derived risk threshold. **(a–d)** Kaplan–Meier (K–M) survival curves for all cases stratified by MVNet-predicted risk using a threshold of 0.1, determined from the Internal-CRCII validation cohort. Curves are shown for Internal-CRCII (a), External-CRCII-1 (b), External-CRCII-2 (c), and TCGA-CRCII (d), respectively. Censored cases are indicated with a ‘+’. Internal-CRCII, internal colorectal cancer stage II cohort; External-CRCII-1, external colorectal cancer stage II cohort 1; External-CRCII-2, external colorectal cancer stage II cohort 2; TCGA-CRCII, TCGA colorectal cancer stage II cohort.
(DOCX)

S15 Fig Similarity assessment before and after heatmap processing. Box plots illustrating similarities before and after heatmap processing in Internal-CRCII **(a)**, External-CRCII-1 **(b)**, External-CRCII-2 **(c)**, and TCGA-CRCII **(d)**. Similarity was measured using the Structural Similarity Index Method (SSIM). The plots display the 25th, 50th (median), and 75th quantiles, as well as the minimum and maximum values. Internal-CRCII, internal colorectal cancer stage II cohort; External-CRCII-1, external colorectal cancer stage II cohort 1; External-CRCII-2, external colorectal cancer stage II cohort 2; TCGA-CRCII, TCGA colorectal cancer stage II cohort.
(DOCX)

S16 Fig Statistical analysis of TLS spatial characteristics. **(a–d)** Frequency distribution histograms of TLS area in Internal-CRCII (a), External-CRCII-1 (b), External-CRCII-2 (c), and TCGA-CRCII (d). **(e–h)** Frequency distribution histograms of the distance between TLS and tumor margin in Internal-CRCII (e), External-CRCII-1 (f), External-CRCII-2 (g), and TCGA-CRCII (h). **(i–l)** Bar graphs showing TLS subtypes in Internal-CRCII (i), External-CRCII-1 (j), External-CRCII-2 (k), and TCGA-CRCII (l). TLS, tertiary lymphoid structure; Agg, aggregates; FL-1, primary follicles; FL-2, secondary follicles; Internal-CRCII, internal colorectal cancer stage II cohort; External-CRCII-1, external colorectal cancer stage II cohort 1; External-CRCII-2, external colorectal cancer stage II cohort 2; TCGA-CRCII, TCGA colorectal cancer stage II cohort.
(DOCX)

S1 Table Classification performance of TNTM. TLS, tertiary lymphoid structure; Internal-CRCII, internal colorectal cancer stage II cohort; External-CRCII-1, external colorectal cancer stage II cohort 1; External-CRCII-2, external colorectal cancer stage II cohort 2.
(DOCX)

S2 Table Classification performance of TLSM. Agg, aggregates; FL-1, primary follicles; FL-2, secondary follicles; Internal-CRCII, internal colorectal cancer stage II cohort; External-CRCII-1, external colorectal cancer stage II cohort 1; External-CRCII-2, external colorectal cancer stage II cohort 2.
(DOCX)

S3 Table Sensitivity and specificity of MVNet at various thresholds across four datasets. Internal-CRCII, internal colorectal cancer stage II cohort; External-CRCII-1, external colorectal cancer stage II cohort 1; External-CRCII-2, external colorectal cancer stage II cohort 2; TCGA-CRCII, TCGA colorectal cancer stage II cohort.
(DOCX)

S4 Table Univariable Cox regression analysis of MVNet-predicted risk in adjuvant chemotherapy (ACT) positive and negative subgroups across multiple cohorts. ACT, adjuvant chemotherapy; Internal-CRCII, internal colorectal cancer stage II cohort; External-CRCII-1, external colorectal cancer stage II cohort 1; External-CRCII-2, external colorectal cancer stage II cohort 2; TCGA-CRCII, TCGA colorectal cancer stage II cohort.
(DOCX)

S5 Table High-risk feature distribution by MVNet risk group and ACT status. MVNet, multi-view network; PNI, perineural invasion; VI, vascular invasion; SRCC, signet-ring cell carcinoma; MAC, mucinous adenocarcinoma; LNS, lymph node sampling; MMR, mismatch repair; ACT, adjuvant chemotherapy; BD3, tumor budding grade 1–3; Internal-CRCII, internal colorectal cancer stage II cohort; External-CRCII-1, external colorectal cancer stage II cohort 1; External-CRCII-2, external colorectal cancer stage II cohort 2.
(DOCX)

S6 Table Interaction analysis between ACT and MVNet risk group using Cox proportional hazards models across cohorts. MVNet, multi-view network; ACT, adjuvant chemotherapy; Internal-CRCII, internal colorectal cancer stage II cohort; External-CRCII-1, external colorectal cancer stage II cohort 1; External-CRCII-2, external colorectal cancer stage II cohort 2.
(DOCX)

S7 Table List of 14 key clinicopathological parameters used for comparison with MVNet in this study. MVNet, multi-view network; PNI, perineural invasion; VI, vascular invasion; SRCC, signet-ring cell carcinoma; MAC, mucinous adenocarcinoma; LNS, lymph node sampling; MMR, mismatch repair.
(DOCX)

S8 Table Comparison of prognostic performance of MVNet across different numbers of randomly sampled tumor slides per patient in a subset of 50 patients. MVNet, multi-view network.
(DOCX)

S9 Table Summary of 226 TLS-related features used in SpB input. TLS, tertiary lymphoid structure.
(DOCX)

S1 Checklist STROBE checklist. This checklist is made available under the Creative Commons Attribution 4.0 (CC BY 4.0) license. The original STROBE checklist can be accessed at https://www.strobe-statement.org/.
(DOCX)

S2 Checklist TRIPOD checklist. This checklist is made available under the Creative Commons Attribution 4.0 (CC BY 4.0) license. The original TRIPOD checklist is available at https://www.tripod-statement.org/.
(DOCX)

## Abbreviations

ACT: adjuvant chemotherapy
AUC: area under the curve
AUROC: area under the receiver operating characteristic curve
CI: confidence interval
CRC: colorectal cancer
DL: deep learning
FC: fully connected
H&E: hematoxylin and eosin
HR: hazard ratio
IHC: Immunohistochemistry
MIL: multiple instance learning
MMR: mismatch repair
MpB: Morphological Branch
NCCN: National Comprehensive Cancer Network
RFS: relapse-free survival
SHAP: Shapley Additive Explanation-based method
SSIM: structural similarity index measure
TLS: tertiary lymphoid structure
TLSM: TLS phenotype classification
TNTM: TLS-normal-tumor tissue classification
WSIs: whole-slide images

## References

[1] Bray F, Laversanne M, Sung H, Ferlay J, Siegel RL, Soerjomataram I, et al. Global cancer statistics 2022: GLOBOCAN estimates of incidence and mortality worldwide for 36 cancers in 185 countries. CA Cancer J Clin. 2024;74(3):229–63. doi: 10.3322/caac.21834 38572751

[2] Böckelman C, Engelmann BE, Kaprio T, Hansen TF, Glimelius B. Risk of recurrence in patients with colon cancer stage II and III: a systematic review and meta-analysis of recent literature. Acta Oncol. 2015;54(1):5–16. doi: 10.3109/0284186X.2014.975839 25430983

[3] Benson AB, Venook AP, Al-Hawary MM, Azad N, Chen Y-J, Ciombor KK, et al. Rectal cancer, version 2.2022, NCCN clinical practice guidelines in oncology. J Natl Compr Canc Netw. 2022;20(10):1139–67. doi: 10.6004/jnccn.2022.0051 36240850

[4] Benson AB, Venook AP, Al-Hawary MM, Arain MA, Chen Y-J, Ciombor KK, et al. Colon cancer, version 2.2021, NCCN clinical practice guidelines in oncology. J Natl Compr Canc Netw. 2021;19(3):329–59. doi: 10.6004/jnccn.2021.0012 33724754

[5] O’Connor ES, Greenblatt DY, LoConte NK, Gangnon RE, Liou J-I, Heise CP, et al. Adjuvant chemotherapy for stage II colon cancer with poor prognostic features. J Clin Oncol. 2011;29(25):3381–8. doi: 10.1200/JCO.2010.34.3426 21788561 PMC3164243

[6] André T, Boni C, Navarro M, Tabernero J, Hickish T, Topham C, et al. Improved overall survival with oxaliplatin, fluorouracil, and leucovorin as adjuvant treatment in stage II or III colon cancer in the MOSAIC trial. J Clin Oncol. 2009;27(19):3109–16. doi: 10.1200/JCO.2008.20.6771 19451431

[7] Kerr RS, Love S, Segelov E, Johnstone E, Falcon B, Hewett P, et al. Adjuvant capecitabine plus bevacizumab versus capecitabine alone in patients with colorectal cancer (QUASAR 2): an open-label, randomised phase 3 trial. Lancet Oncol. 2016;17(11):1543–57. doi: 10.1016/S1470-2045(16)30172-3 27660192

[8] Quasar Collaborative Group, Gray R, Barnwell J, McConkey C, Hills RK, Williams NS, et al. Adjuvant chemotherapy versus observation in patients with colorectal cancer: a randomised study. Lancet. 2007;370(9604):2020–9. doi: 10.1016/S0140-6736(07)61866-2 18083404

[9] Merok MA, Ahlquist T, Røyrvik EC, Tufteland KF, Hektoen M, Sjo OH, et al. Microsatellite instability has a positive prognostic impact on stage II colorectal cancer after complete resection: results from a large, consecutive Norwegian series. Ann Oncol. 2013;24(5):1274–82. doi: 10.1093/annonc/mds614 23235802 PMC3629894

[10] Schetter AJ, Nguyen GH, Bowman ED, Mathé EA, Yuen ST, Hawkes JE, et al. Association of inflammation-related and microRNA gene expression with cancer-specific mortality of colon adenocarcinoma. Clin Cancer Res. 2009;15(18):5878–87. doi: 10.1158/1078-0432.CCR-09-0627 19737943 PMC2745503

[11] Reinert T, Henriksen TV, Christensen E, Sharma S, Salari R, Sethi H, et al. Analysis of plasma cell-free DNA by ultradeep sequencing in patients with stages I to III colorectal cancer. JAMA Oncol. 2019;5(8):1124–31. doi: 10.1001/jamaoncol.2019.0528 31070691 PMC6512280

[12] Luo X-J, Zhao Q, Liu J, Zheng J-B, Qiu M-Z, Ju H-Q, et al. Novel genetic and epigenetic biomarkers of prognostic and predictive significance in stage II/III colorectal cancer. Mol Ther. 2021;29(2):587–96. doi: 10.1016/j.ymthe.2020.12.017 33333293 PMC7854353

[13] Toh JWT, Phan K, Reza F, Chapuis P, Spring KJ. Rate of dissemination and prognosis in early and advanced stage colorectal cancer based on microsatellite instability status: systematic review and meta-analysis. Int J Colorectal Dis. 2021;36(8):1573–96. doi: 10.1007/s00384-021-03874-1 33604737

[14] Zhou D, Tian F, Tian X, Sun L, Huang X, Zhao F, et al. Diagnostic evaluation of a deep learning model for optical diagnosis of colorectal cancer. Nat Commun. 2020;11(1):2961. doi: 10.1038/s41467-020-16777-6 32528084 PMC7289893

[15] Yamashita R, Long J, Longacre T, Peng L, Berry G, Martin B, et al. Deep learning model for the prediction of microsatellite instability in colorectal cancer: a diagnostic study. Lancet Oncol. 2021;22(1):132–41. doi: 10.1016/S1470-2045(20)30535-0 33387492

[16] Skrede O-J, De Raedt S, Kleppe A, Hveem TS, Liestøl K, Maddison J, et al. Deep learning for prediction of colorectal cancer outcome: a discovery and validation study. Lancet. 2020;395(10221):350–60. doi: 10.1016/S0140-6736(19)32998-8 32007170

[17] Jiang X, Hoffmeister M, Brenner H, Muti HS, Yuan T, Foersch S, et al. End-to-end prognostication in colorectal cancer by deep learning: a retrospective, multicentre study. Lancet Digit Health. 2024;6(1):e33–43. doi: 10.1016/S2589-7500(23)00208-X 38123254

[18] Masuda T, Tanaka N, Takamatsu K, Hakozaki K, Takahashi R, Anno T, et al. Unique characteristics of tertiary lymphoid structures in kidney clear cell carcinoma: prognostic outcome and comparison with bladder cancer. J Immunother Cancer. 2022;10(3):e003883. doi: 10.1136/jitc-2021-003883 35314433 PMC8938705

[19] Liang J, Zhang W, Yang J, Wu M, Dai Q, Yin H, et al. Deep learning supported discovery of biomarkers for clinical prognosis of liver cancer. Nat Mach Intell. 2023;5(4):408–20. doi: 10.1038/s42256-023-00635-3

[20] Vahadane A, Peng T, Sethi A, Albarqouni S, Wang L, Baust M, et al. Structure-preserving color normalization and sparse stain separation for histological images. IEEE Trans Med Imaging. 2016;35(8):1962–71. doi: 10.1109/TMI.2016.2529665 27164577

[21] Lu MY, Williamson DFK, Chen TY, Chen RJ, Barbieri M, Mahmood F. Data-efficient and weakly supervised computational pathology on whole-slide images. Nat Biomed Eng. 2021;5(6):555–70. doi: 10.1038/s41551-020-00682-w 33649564 PMC8711640

[22] Wang X, Yang S, Zhang J, Wang M, Zhang J, Yang W, et al. Transformer-based unsupervised contrastive learning for histopathological image classification. Med Image Anal. 2022;81:102559. doi: 10.1016/j.media.2022.102559 35952419

[23] Yao J, Zhu X, Jonnagaddala J, Hawkins N, Huang J. Whole slide images based cancer survival prediction using attention guided deep multiple instance learning networks. Med Image Anal. 2020;65:101789. doi: 10.1016/j.media.2020.101789 32739769

[24] Foersch S, Glasner C, Woerl A-C, Eckstein M, Wagner D-C, Schulz S, et al. Multistain deep learning for prediction of prognosis and therapy response in colorectal cancer. Nat Med. 2023;29(2):430–9. doi: 10.1038/s41591-022-02134-1 36624314

[25] Frederick L, Page DL, Fleming ID, Fritz AG, Balch CM, Haller DG, et al. AJCC cancer staging manual. Springer Science & Business Media; 2002.

[26] Pham HHN, Futakuchi M, Bychkov A, Furukawa T, Kuroda K, Fukuoka J. Detection of lung cancer lymph node metastases from whole-slide histopathologic images using a two-step deep learning approach. Am J Pathol. 2019;189(12):2428–39. doi: 10.1016/j.ajpath.2019.08.014 31541645

[27] Larbi A, Nyarko E, Iddi S. Evaluation of optimal strategies for breast cancer screening in Ghana: a simulation study based on a continuous tumor growth model. PLoS One. 2025;20(6):e0323485. doi: 10.1371/journal.pone.0323485 40526619 PMC12173189

[28] Gül M. A novel local binary patterns-based approach and proposed CNN model to diagnose breast cancer by analyzing histopathology images. IEEE Access. 2025;13:39610–20. doi: 10.1109/access.2025.3545052

[29] Lundberg SM, Lee SI. A unified approach to interpreting model predictions. Adv Neural Inf Process Syst 2017;30.

[30] Gao P, Xiao Q, Tan H, Song J, Fu Y, Xu J, et al. Interpretable multi-modal artificial intelligence model for predicting gastric cancer response to neoadjuvant chemotherapy. Cell Rep Med. 2024;5(12):101848. doi: 10.1016/j.xcrm.2024.101848 39637859 PMC11722130

[31] Garriga R, Buda TS, Guerreiro J, Omaña Iglesias J, Estella Aguerri I, Matić A. Combining clinical notes with structured electronic health records enhances the prediction of mental health crises. Cell Rep Med. 2023;4(11):101260. doi: 10.1016/j.xcrm.2023.101260 37913776 PMC10694623

[32] Zhang Y, Liu G, Zeng Q, Wu W, Lei K, Zhang C, et al. CCL19-producing fibroblasts promote tertiary lymphoid structure formation enhancing anti-tumor IgG response in colorectal cancer liver metastasis. Cancer Cell. 2024;42(8):1370-1385.e9. doi: 10.1016/j.ccell.2024.07.006 39137726

[33] Kornprat P, Pollheimer MJ, Lindtner RA, Schlemmer A, Rehak P, Langner C. Value of tumor size as a prognostic variable in colorectal cancer: a critical reappraisal. Am J Clin Oncol. 2011;34(1):43–9. doi: 10.1097/COC.0b013e3181cae8dd 20101166

[34] Saha S, Shaik M, Johnston G, Saha SK, Berbiglia L, Hicks M, et al. Tumor size predicts long-term survival in colon cancer: an analysis of the National Cancer Data Base. Am J Surg. 2015;209(3):570–4. doi: 10.1016/j.amjsurg.2014.12.008 25601557

[35] Trajkovski G, Ognjenovic L, Karadzov Z, Jota G, Hadzi-Manchev D, Kostovski O, et al. Tertiary lymphoid structures in colorectal cancers and their prognostic value. Open Access Maced J Med Sci. 2018;6(10):1824–8. doi: 10.3889/oamjms.2018.341 30455756 PMC6236051

[36] Parent P, Cohen R, Rassy E, Svrcek M, Taieb J, André T, et al. A comprehensive overview of promising biomarkers in stage II colorectal cancer. Cancer Treat Rev. 2020;88:102059. doi: 10.1016/j.ctrv.2020.102059 32622273

[37] Agesen TH, Sveen A, Merok MA, Lind GE, Nesbakken A, Skotheim RI, et al. ColoGuideEx: a robust gene classifier specific for stage II colorectal cancer prognosis. Gut. 2012;61(11):1560–7. doi: 10.1136/gutjnl-2011-301179 22213796

[38] Caputo F, Santini C, Bardasi C, Cerma K, Casadei-Gardini A, Spallanzani A, et al. BRAF-mutated colorectal cancer: clinical and molecular insights. Int J Mol Sci. 2019;20(21):5369. doi: 10.3390/ijms20215369 31661924 PMC6861966

[39] Zhu G, Pei L, Xia H, Tang Q, Bi F. Role of oncogenic KRAS in the prognosis, diagnosis and treatment of colorectal cancer. Mol Cancer. 2021;20(1):143. doi: 10.1186/s12943-021-01441-4 34742312 PMC8571891

[40] Kleppe A, Skrede O-J, De Raedt S, Hveem TS, Askautrud HA, Jacobsen JE, et al. A clinical decision support system optimising adjuvant chemotherapy for colorectal cancers by integrating deep learning and pathological staging markers: a development and validation study. Lancet Oncol. 2022;23(9):1221–32. doi: 10.1016/S1470-2045(22)00391-6 35964620

[41] Kundu S. AI in medicine must be explainable. Nat Med. 2021;27(8):1328. doi: 10.1038/s41591-021-01461-z 34326551

[42] Collins GS, Moons KGM. Reporting of artificial intelligence prediction models. Lancet. 2019;393(10181):1577–9. doi: 10.1016/S0140-6736(19)30037-6 31007185

[43] Wulczyn E, Steiner DF, Moran M, Plass M, Reihs R, Tan F, et al. Interpretable survival prediction for colorectal cancer using deep learning. NPJ Digit Med. 2021;4(1):71. doi: 10.1038/s41746-021-00427-2 33875798 PMC8055695

[44] Reitsam NG, Grosser B, Steiner DF, Grozdanov V, Wulczyn E, L’Imperio V, et al. Converging deep learning and human-observed tumor-adipocyte interaction as a biomarker in colorectal cancer. Commun Med (Lond). 2024;4(1):163. doi: 10.1038/s43856-024-00589-6 39147895 PMC11327259

[45] Schumacher TN, Thommen DS. Tertiary lymphoid structures in cancer. Science. 2022;375(6576):eabf9419. doi: 10.1126/science.abf9419 34990248

[46] Sautès-Fridman C, Petitprez F, Calderaro J, Fridman WH. Tertiary lymphoid structures in the era of cancer immunotherapy. Nat Rev Cancer. 2019;19(6):307–25. doi: 10.1038/s41568-019-0144-6 31092904

[47] Li Z, Jiang Y, Li B, Han Z, Shen J, Xia Y, et al. Development and validation of a machine learning model for detection and classification of tertiary lymphoid structures in gastrointestinal cancers. JAMA Netw Open. 2023;6(1):e2252553. doi: 10.1001/jamanetworkopen.2022.52553 36692877 PMC10408275

[48] Posch F, Silina K, Leibl S, Mündlein A, Moch H, Siebenhüner A, et al. Maturation of tertiary lymphoid structures and recurrence of stage II and III colorectal cancer. Oncoimmunology. 2017;7(2):e1378844. doi: 10.1080/2162402X.2017.1378844 29416939 PMC5798199

[49] Ren Z, Wang S, Zhang Y. Weakly supervised machine learning. CAAI Trans on Intel Tech. 2023;8(3):549–80. doi: 10.1049/cit2.12216

[50] Chung PC, Yang WJ, Wu TH, Huang CR, Hsu YY, editors. Emerging research directions of deep learning for pathology image analysis. 2022 IEEE Biomedical Circuits and Systems Conference (BioCAS); 2022 Oct 13–; 2022.

[51] Frénay B, Verleysen M. Classification in the presence of label noise: a survey. IEEE Trans Neural Netw Learn Syst. 2014;25(5):845–69. doi: 10.1109/TNNLS.2013.2292894 24808033

[52] Campanella G, Hanna MG, Geneslaw L, Miraflor A, Werneck Krauss Silva V, Busam KJ, et al. Clinical-grade computational pathology using weakly supervised deep learning on whole slide images. Nat Med. 2019;25(8):1301–9. doi: 10.1038/s41591-019-0508-1 31308507 PMC7418463

[53] Zeng W. Image data augmentation techniques based on deep learning: a survey. Math Biosci Eng. 2024;21(6):6190–224. doi: 10.3934/mbe.2024272 39176424

[54] Deng R, Cui C, Remedios LW, Bao S, Womick RM, Chiron S, et al. Cross-scale multi-instance learning for pathological image diagnosis. Med Image Anal. 2024;94:103124. doi: 10.1016/j.media.2024.103124 38428271 PMC11016375

[55] Lee Y, Park JH, Oh S, Shin K, Sun J, Jung M, et al. Derivation of prognostic contextual histopathological features from whole-slide images of tumours via graph deep learning. Nat Biomed Eng. 2022;6(12):1452–66. doi: 10.1038/s41551-022-00923-0 35982331

[56] Wagner SJ, Reisenbüchler D, West NP, Niehues JM, Zhu J, Foersch S, et al. Transformer-based biomarker prediction from colorectal cancer histology: a large-scale multicentric study. Cancer Cell. 2023;41(9):1650-1661.e4. doi: 10.1016/j.ccell.2023.08.002 37652006 PMC10507381

