# Supplementary material for: Multiview deep-learning-enabled histopathology for prognostic and therapeutic stratification in stage II colorectal cancer: A retrospective multicenter study
Source: PLoS Med. 2026 Jan 13;23(1):e1004614. doi: 10.1371/journal.pmed.1004614 (PMC12801286; doi:10.1371/journal.pmed.1004614)
Supplement: S1 Fig — CONSORT diagrams for data filtering and baseline characteristics of study participants in Internal-CRCII (a), External-CRCII-1 (b), External-CRCII-2 (c), and TCGA-CRCII (d), respectively. CRC, colorectal cancer; WSI, whole slide image; RFS, relapse-free survival; MMR, mismatch repair; Internal-CRCII, internal colorectal cancer stage II cohort; External-CRCII-1, external colorectal cancer stage II cohort 1; External-CRCII-2, external colorectal cancer stage II cohort 2; TCGA-CRCII, TCGA colorectal cancer stage II cohort. (DOCX) [file pmed.1004614.s001.docx]

**S1 Fig. CONSORT diagrams and baselines of study participants.**

CONSORT diagrams for data filtering and baseline characteristics of study participants in Internal-CRCII (a), External-CRCII-1 (b), External-CRCII-2 (c), and TCGA-CRCII (d), respectively. CRC, colorectal cancer; WSI, whole slide image; RFS, relapse-free survival; MMR, mismatch repair; Internal-CRCII, internal colorectal cancer stage II cohort; External-CRCII-1, external colorectal cancer stage II cohort 1; External-CRCII-2, external colorectal cancer stage II cohort 2; TCGA-CRCII, TCGA colorectal cancer stage II cohort.
