# Supplementary material for: Multiview deep-learning-enabled histopathology for prognostic and therapeutic stratification in stage II colorectal cancer: A retrospective multicenter study
Source: PLoS Med. 2026 Jan 13;23(1):e1004614. doi: 10.1371/journal.pmed.1004614 (PMC12801286; doi:10.1371/journal.pmed.1004614)
Supplement: S2 Fig — Representative image patches from the cluster with the highest patch count in the no-relapse group. These patches predominantly display dense lymphoid aggregates and follicle-like structures, consistent with the morphology of tertiary lymphoid structures (TLSs). Each patch corresponds to a tissue area of 128 μm × 128 μm. (DOCX) [file pmed.1004614.s002.docx]

**S2 Fig. Representative patches from the most abundant cluster in the no-relapse group.**

Representative image patches from the cluster with the highest patch count in the no-relapse group. These patches predominantly display dense lymphoid aggregates and follicle-like structures, consistent with the morphology of tertiary lymphoid structures (TLSs). Each patch corresponds to a tissue area of 128 μm × 128 μm.
