# Supplementary material for: Multiview deep-learning-enabled histopathology for prognostic and therapeutic stratification in stage II colorectal cancer: A retrospective multicenter study
Source: PLoS Med. 2026 Jan 13;23(1):e1004614. doi: 10.1371/journal.pmed.1004614 (PMC12801286; doi:10.1371/journal.pmed.1004614)
Supplement: S3 Fig — (a) Model architecture of MVNet and MMF, illustrating the integration of SpB, MpB and clinical parameters for multi-view analysis. (b) Model architecture of the MpB branch, focusing on morphological feature extraction within the MVNet framework. Fc(x) represents a fully connected layer with an output dimension x. WSI, whole slide image; MVNet, multi-view network; TLS, tertiary lymphoid structure; ACT, adjuvant chemotherapy; MpB, morphological feature branch; SpB, spatial feature branch; TLSM, TLS subtyping model; MMF, multi-modal fusion. (DOCX) [file pmed.1004614.s003.docx]

**S3 Fig. Overview of MVNet, MMF and MpB branch.**

(a) Model architecture of MVNet and MMF, illustrating the integration of SpB, MpB and clinical parameters for multi-view analysis. (b) Model architecture of the MpB branch, focusing on morphological feature extraction within the MVNet framework. Fc(x) represents a fully connected layer with an output dimension x.

WSI, whole slide image; MVNet, multi-view network; TLS, tertiary lymphoid structure; ACT, adjuvant chemotherapy; MpB, morphological feature branch; SpB, spatial feature branch; TLSM, TLS subtyping model; MMF, multi-modal fusion.
