# Supplementary material for: Multiview deep-learning-enabled histopathology for prognostic and therapeutic stratification in stage II colorectal cancer: A retrospective multicenter study
Source: PLoS Med. 2026 Jan 13;23(1):e1004614. doi: 10.1371/journal.pmed.1004614 (PMC12801286; doi:10.1371/journal.pmed.1004614)
Supplement: S4 Fig — Confusion matrices illustrating the performance of MVNet predictions for Internal-CRCII (a), External-CRCII-1 (b), External-CRCII-2 (c), and TCGA-CRCII (d) cohorts. The chi-squared test was employed to assess performance. Statistical significance is denoted as follows: ns, p > 0.05; *p ≤ 0.05; **p ≤ 0.01; ***p ≤ 0.001; ****p ≤ 0.0001. ns, not significant; Internal-CRCII, internal colorectal cancer stage II cohort; External-CRCII-1, external colorectal cancer stage II cohort 1; External-CRCII-2, external colorectal cancer stage II cohort 2; TCGA-CRCII, TCGA colorectal cancer stage II cohort. (DOCX) [file pmed.1004614.s004.docx]

**S4 Fig. Confusion matrices of MVNet predictions.**

Confusion matrices illustrating the performance of MVNet predictions for Internal-CRCII (a), External-CRCII-1 (b), External-CRCII-2 (c), and TCGA-CRCII (d) cohorts. The chi-squared test was employed to assess performance. Statistical significance is denoted as follows: ns, p > 0.05; *p ≤ 0.05; **p ≤ 0.01; ***p ≤ 0.001; ****p ≤ 0.0001. ns, not significant; Internal-CRCII, internal colorectal cancer stage II cohort; External-CRCII-1, external colorectal cancer stage II cohort 1; External-CRCII-2, external colorectal cancer stage II cohort 2; TCGA-CRCII, TCGA colorectal cancer stage II cohort.
