# Supplementary material for: Multiview deep-learning-enabled histopathology for prognostic and therapeutic stratification in stage II colorectal cancer: A retrospective multicenter study
Source: PLoS Med. 2026 Jan 13;23(1):e1004614. doi: 10.1371/journal.pmed.1004614 (PMC12801286; doi:10.1371/journal.pmed.1004614)
Supplement: S5 Fig — (a) Comparison of AUROCs among WSINet, two branches, and MVNet on the Internal-CRCII cohort. (b–d) Comparison of AUROCs among the two branches and MVNet on the External-CRCII-1 (b), External-CRCII-2 (c), and TCGA-CRCII (d) cohorts, respectively. DeLong’s test was used for statistical assessment. Significance is denoted as follows: ns, p > 0.05; *p ≤ 0.05; **p ≤ 0.01; ***p ≤ 0.001; ****p ≤ 0.0001. ns, not significant; SpB, spatial feature branch; MpB, morphological feature branch; MVNet, multi-view network; Internal-CRCII, internal colorectal cancer stage II cohort; External-CRCII-1, external colorectal cancer stage II cohort 1; External-CRCII-2, external colorectal cancer stage II cohort 2; TCGA-CRCII, TCGA colorectal cancer stage II cohort. (DOCX) [file pmed.1004614.s005.docx]

**S5 Fig. Comparison of MVNet predictive performance.**

(a) Comparison of AUROCs among WSINet, two branches, and MVNet on the Internal-CRCII cohort. (b-d) Comparison of AUROCs among the two branches and MVNet on the External-CRCII-1 (b), External-CRCII-2 (c), and TCGA-CRCII (d) cohorts, respectively. DeLong's test was used for statistical assessment. Significance is denoted as follows: ns, p > 0.05; *p ≤ 0.05; **p ≤ 0.01; ***p ≤ 0.001; ****p ≤ 0.0001. ns, not significant; SpB, spatial feature branch; MpB, morphological feature branch; MVNet, multi-view network; Internal-CRCII, internal colorectal cancer stage II cohort; External-CRCII-1, external colorectal cancer stage II cohort 1; External-CRCII-2, external colorectal cancer stage II cohort 2; TCGA-CRCII, TCGA colorectal cancer stage II cohort.
