# Supplementary material for: Multiview deep-learning-enabled histopathology for prognostic and therapeutic stratification in stage II colorectal cancer: A retrospective multicenter study
Source: PLoS Med. 2026 Jan 13;23(1):e1004614. doi: 10.1371/journal.pmed.1004614 (PMC12801286; doi:10.1371/journal.pmed.1004614)
Supplement: S6 Fig — (a) Univariate Cox regression analysis for Internal-CRCII. (b) Multivariate Cox regression analysis for Internal-CRCII. (c, d) Univariate (c) and Multivariate (d) Cox regression analyses for External-CRCII-1. (e, f) Univariate (e) and Multivariate (f) Cox regression analyses for External-CRCII-2. (g, h) Univariate (g) and Multivariate (h) Cox regression analyses for TCGA-CRCII. Statistical significance was calculated using the Wald test. MVNet, multi-view network; PNI, perineural invasion; VI, vascular invasion; SRCC, signet-ring cell carcinoma; MAC, mucinous adenocarcinoma; LNS, lymph node sampling; MMR, mismatch repair; ACT, adjuvant chemotherapy; BD3, tumor budding grade 1–3; Internal-CRCII, internal colorectal cancer stage II cohort; External-CRCII-1, external colorectal cancer stage II cohort 1; External-CRCII-2, external colorectal cancer stage II cohort 2. (DOCX) [file pmed.1004614.s006.docx]

**S6 Fig. Cox regression analysis of MVNet and clinicopathological parameters.**

(a) Univariate Cox regression analysis for Internal-CRCII. (b) Multivariate Cox regression analysis for Internal-CRCII. (c-d) Univariate (c) and Multivariate (d) Cox regression analyses for External-CRCII-1. (e-f) Univariate (e) and Multivariate (f) Cox regression analyses for External-CRCII-2. (g-h) Univariate (g) and Multivariate (h) Cox regression analyses for TCGA-CRCII. Statistical significance was calculated using the Wald test. MVNet, multi-view network; PNI, perineural invasion; VI, vascular invasion; SRCC, signet-ring cell carcinoma; MAC, mucinous adenocarcinoma; LNS, lymph node sampling; MMR, mismatch repair; ACT, adjuvant chemotherapy; BD3, tumor budding grade 1-3; Internal-CRCII, internal colorectal cancer stage II cohort; External-CRCII-1, external colorectal cancer stage II cohort 1; External-CRCII-2, external colorectal cancer stage II cohort 2.
