# Supplementary material for: Multiview deep-learning-enabled histopathology for prognostic and therapeutic stratification in stage II colorectal cancer: A retrospective multicenter study
Source: PLoS Med. 2026 Jan 13;23(1):e1004614. doi: 10.1371/journal.pmed.1004614 (PMC12801286; doi:10.1371/journal.pmed.1004614)
Supplement: S7 Fig — (a–d) Correlation maps illustrating statistically significant clinical parameters and MVNet score for Internal-CRCII (a), External-CRCII-1 (b), External-CRCII-2 (c), and TCGA-CRCII (d), respectively. (e–h) Nomograms predicting the proportion of cases with relapse-free survival post-operation for Internal-CRCII (e), External-CRCII-1 (f), External-CRCII-2 (g), and TCGA-CRCII (h), respectively. (i–l) Calibration plots of the MVNet-clinic linear model comparing observed and predicted 5-year outcomes for Internal-CRCII (i), External-CRCII-1 (j), External-CRCII-2 (k), and TCGA-CRCII (l), respectively. Perfect performance is represented by points along the 45-degree line. MVNet, multi-view network; PNI, perineural invasion; SRCC, signet-ring cell carcinoma; MAC, mucinous adenocarcinoma; LNS, lymph node sampling; MMR, mismatch repair; RFS, relapse-free survival; Internal-CRCII, internal colorectal cancer stage II cohort; External-CRCII-1, external colorectal cancer stage II cohort 1; External-CRCII-2, external colorectal cancer stage II cohort 2; TCGA-CRCII, TCGA colorectal cancer stage II cohort. (DOCX) [file pmed.1004614.s007.docx]

**S7 Fig. Correlation maps and nomograms of MVNet and clinicopathological parameters.**

(a-d) Correlation maps illustrating statistically significant clinical parameters and MVNet score for Internal-CRCII (a), External-CRCII-1 (b), External-CRCII-2 (c), and TCGA-CRCII (d), respectively. (e-h) Nomograms predicting the proportion of cases with relapse-free survival post-operation for Internal-CRCII (e), External-CRCII-1 (f), External-CRCII-2 (g), and TCGA-CRCII (h), respectively. (i-l) Calibration plots of the MVNet-clinic linear model comparing observed and predicted 5-year outcomes for Internal-CRCII (i), External-CRCII-1 (j), External-CRCII-2 (k), and TCGA-CRCII (l), respectively. Perfect performance is represented by points along the 45-degree line. MVNet, multi-view network; PNI, perineural invasion; SRCC, signet-ring cell carcinoma; MAC, mucinous adenocarcinoma; LNS, lymph node sampling; MMR, mismatch repair; RFS, relapse-free survival; Internal-CRCII, internal colorectal cancer stage II cohort; External-CRCII-1, external colorectal cancer stage II cohort 1; External-CRCII-2, external colorectal cancer stage II cohort 2; TCGA-CRCII, TCGA colorectal cancer stage II cohort.
