# Supplementary material for: Multiview deep-learning-enabled histopathology for prognostic and therapeutic stratification in stage II colorectal cancer: A retrospective multicenter study
Source: PLoS Med. 2026 Jan 13;23(1):e1004614. doi: 10.1371/journal.pmed.1004614 (PMC12801286; doi:10.1371/journal.pmed.1004614)
Supplement: S8 Fig — (a–c) Receiver operating characteristic (ROC) curves of the clinicopathological feature–only model in Internal-CRCII (a), External-CRCII-1 (b), and External-CRCII-2 (c) cohorts, respectively. Internal-CRCII, internal colorectal cancer stage II cohort; External-CRCII-1, external colorectal cancer stage II cohort 1; External-CRCII-2, external colorectal cancer stage II cohort 2. (DOCX) [file pmed.1004614.s008.docx]

**S8 Fig. Predictive performance of clinicopathological feature–only model across datasets.**

(a–c) Receiver operating characteristic (ROC) curves of the clinicopathological feature–only model in Internal-CRCII (a), External-CRCII-1 (b), and External-CRCII-2 (c) cohorts, respectively. Internal-CRCII, internal colorectal cancer stage II cohort; External-CRCII-1, external colorectal cancer stage II cohort 1; External-CRCII-2, external colorectal cancer stage II cohort 2.
