# Supplementary material for: Multiview deep-learning-enabled histopathology for prognostic and therapeutic stratification in stage II colorectal cancer: A retrospective multicenter study
Source: PLoS Med. 2026 Jan 13;23(1):e1004614. doi: 10.1371/journal.pmed.1004614 (PMC12801286; doi:10.1371/journal.pmed.1004614)
Supplement: S9 Fig — (a–c) Kaplan–Meier (K–M) survival curves for patients who received adjuvant chemotherapy (ACT+, ACT+ subgroup) from Internal-CRCII (a), External-CRCII-1 (b), and External-CRCII-2 (c), stratified by the predicted risk from MVNet. (d–f) Kaplan–Meier (K–M) survival curves for patients who did not receive adjuvant chemotherapy (ACT−, ACT− subgroup) from Internal-CRCII (d), External-CRCII-1 (e), and External-CRCII-2 (f), stratified by the predicted risk from MVNet. Censors are indicated with a ‘+’. The log-rank test was used to calculate statistical significance. Statistical significance is indicated as follows: ns, p > 0.05; *p ≤ 0.05; **p ≤ 0.01; ***p ≤ 0.001; ****p ≤ 0.0001. ns, not significant; ACT, adjuvant chemotherapy; Internal-CRCII, internal colorectal cancer stage II cohort; External-CRCII-1, external colorectal cancer stage II cohort 1; External-CRCII-2, external colorectal cancer stage II cohort 2. (DOCX) [file pmed.1004614.s009.docx]

**S9 Fig. Prognostic performance of SurvFinder stratified by adjuvant chemotherapy receipt.**

(a-c) Kaplan-Meier (K-M) survival curves for patients who received adjuvant chemotherapy (ACT+, ACT+ subgroup) from Internal-CRCII (a), External-CRCII-1 (b), and External-CRCII-2 (c), stratified by the predicted risk from MVNet. (d-f) Kaplan-Meier (K-M) survival curves for patients who did not receive adjuvant chemotherapy (ACT–, ACT– subgroup) from Internal-CRCII (d), External-CRCII-1 (e), and External-CRCII-2 (f), stratified by the predicted risk from MVNet. Censors are indicated with a '+'. The log-rank test was used to calculate statistical significance. Statistical significance is indicated as follows: ns, p > 0.05; *p ≤ 0.05; **p ≤ 0.01; ***p ≤ 0.001; ****p ≤ 0.0001. ns, not significant; ACT, adjuvant chemotherapy; Internal-CRCII, internal colorectal cancer stage II cohort; External-CRCII-1, external colorectal cancer stage II cohort 1; External-CRCII-2, external colorectal cancer stage II cohort 2.
