# Supplementary material for: Multiview deep-learning-enabled histopathology for prognostic and therapeutic stratification in stage II colorectal cancer: A retrospective multicenter study
Source: PLoS Med. 2026 Jan 13;23(1):e1004614. doi: 10.1371/journal.pmed.1004614 (PMC12801286; doi:10.1371/journal.pmed.1004614)
Supplement: S10 Fig — (a, b) Typical WSI examples from the ‘No relapse’ group, showing SurvFinder interpretation results. Each includes the original H&E-stained WSI image (left), the WSI image overlaid with the SegNet-predicted heatmap (middle), and descriptions of the typical TLSs (right). (c, d) Typical WSI examples from the ‘Relapse’ group, following the same format. Each group includes two cases correctly predicted as low-risk and high-risk by SurvFinder, respectively. The scale bar in each image represents a length of 5 mm. TLS, tertiary lymphoid structure; SegNet, segmentation network. (DOCX) [file pmed.1004614.s010.docx]

**S10 Fig. SurvFinder interpretation on WSIs from “No relapse” and “Relapse” Groups.**

(a-b) Typical WSI examples from the 'No relapse' group, showing SurvFinder interpretation results. Each includes the original H&E-stained WSI image (left), the WSI image overlaid with the SegNet-predicted heatmap (middle), and descriptions of the typical TLSs (right). (c-d) Typical WSI examples from the 'Relapse' group, following the same format. Each group includes two cases correctly predicted as low-risk and high-risk by SurvFinder, respectively. The scale bar in each image represents a length of 5 mm. TLS, tertiary lymphoid structure; SegNet, segmentation network.
