# Supplementary material for: Multiview deep-learning-enabled histopathology for prognostic and therapeutic stratification in stage II colorectal cancer: A retrospective multicenter study
Source: PLoS Med. 2026 Jan 13;23(1):e1004614. doi: 10.1371/journal.pmed.1004614 (PMC12801286; doi:10.1371/journal.pmed.1004614)
Supplement: S11 Fig — Representative images of TLS classifications (Agg, FL-1, and FL-2) and immunohistochemical patterns stained with CD20, CD21, and CD10. The scale bar in each image represents 250 µm. Agg, aggregates; FL-1, primary follicles; FL-2, secondary follicles. (DOCX) [file pmed.1004614.s011.docx]

**S11 Fig. Classifications of TLS determined by immunohistochemical staining.**

Representative images of TLS classifications (Agg, FL-1, and FL-2) and immunohistochemical patterns stained with CD20, CD21, and CD10. The scale bar in each image represents 250 µm. Agg, aggregates; FL-1, primary follicles; FL-2, secondary follicles.
