# Supplementary material for: Multiview deep-learning-enabled histopathology for prognostic and therapeutic stratification in stage II colorectal cancer: A retrospective multicenter study
Source: PLoS Med. 2026 Jan 13;23(1):e1004614. doi: 10.1371/journal.pmed.1004614 (PMC12801286; doi:10.1371/journal.pmed.1004614)
Supplement: S12 Fig — Comparative visualization of tissue regions before and after background removal. (a) and (c) show tile regions after applying Otsu’s method, effectively excluding non-tissue white areas. (b) and (d) display the corresponding original whole-slide image thumbnails prior to processing. (DOCX) [file pmed.1004614.s012.docx]

**S12 Fig. Visualization of white background removal using Otsu’s method.**

Comparative visualization of tissue regions before and after background removal. (a) and (c) show tile regions after applying Otsu’s method, effectively excluding non-tissue white areas. (b) and (d) display the corresponding original whole-slide image thumbnails prior to processing.
