# Supplementary material for: Multiview deep-learning-enabled histopathology for prognostic and therapeutic stratification in stage II colorectal cancer: A retrospective multicenter study
Source: PLoS Med. 2026 Jan 13;23(1):e1004614. doi: 10.1371/journal.pmed.1004614 (PMC12801286; doi:10.1371/journal.pmed.1004614)
Supplement: S13 Fig — Predictive performance comparison on the External-CRCII-2 dataset. (a) AUROC curve based on stain-normalized images processed using the Vahadane method. (b) AUROC curve based on the corresponding unstained (original) images. The stained group consistently exhibited higher predictive accuracy than the unstained group. (DOCX) [file pmed.1004614.s013.docx]

**S13 Fig. Comparison of predictive performance between stain-normalized and unstained images in External-CRCII-2.**

Predictive performance comparison on the External-CRCII-2 dataset. (a) AUROC curve based on stain-normalized images processed using the Vahadane method. (b) AUROC curve based on the corresponding unstained (original) images. The stained group consistently exhibited higher predictive accuracy than the unstained group.
