# Supplementary material for: Multiview deep-learning-enabled histopathology for prognostic and therapeutic stratification in stage II colorectal cancer: A retrospective multicenter study
Source: PLoS Med. 2026 Jan 13;23(1):e1004614. doi: 10.1371/journal.pmed.1004614 (PMC12801286; doi:10.1371/journal.pmed.1004614)
Supplement: S14 Fig — (a–d) Kaplan–Meier (K–M) survival curves for all cases stratified by MVNet-predicted risk using a threshold of 0.1, determined from the Internal-CRCII validation cohort. Curves are shown for Internal-CRCII (a), External-CRCII-1 (b), External-CRCII-2 (c), and TCGA-CRCII (d), respectively. Censored cases are indicated with a ‘+’. Internal-CRCII, internal colorectal cancer stage II cohort; External-CRCII-1, external colorectal cancer stage II cohort 1; External-CRCII-2, external colorectal cancer stage II cohort 2; TCGA-CRCII, TCGA colorectal cancer stage II cohort. (DOCX) [file pmed.1004614.s014.docx]

**S14 Fig. Prognostic performance of SurvFinder using an Internal-CRCII-derived risk threshold.**

(a-d) Kaplan-Meier (K-M) survival curves for all cases stratified by MVNet-predicted risk using a threshold of 0.1, determined from the Internal-CRCII validation cohort. Curves are shown for Internal-CRCII (a), External-CRCII-1 (b), External-CRCII-2 (c), and TCGA-CRCII (d), respectively. Censored cases are indicated with a '+'. Internal-CRCII, internal colorectal cancer stage II cohort; External-CRCII-1, external colorectal cancer stage II cohort 1; External-CRCII-2, external colorectal cancer stage II cohort 2; TCGA-CRCII, TCGA colorectal cancer stage II cohort.
