# Supplementary material for: Multiview deep-learning-enabled histopathology for prognostic and therapeutic stratification in stage II colorectal cancer: A retrospective multicenter study
Source: PLoS Med. 2026 Jan 13;23(1):e1004614. doi: 10.1371/journal.pmed.1004614 (PMC12801286; doi:10.1371/journal.pmed.1004614)
Supplement: S15 Fig — Box plots illustrating similarities before and after heatmap processing in Internal-CRCII (a), External-CRCII-1 (b), External-CRCII-2 (c), and TCGA-CRCII (d). Similarity was measured using the Structural Similarity Index Method (SSIM). The plots display the 25th, 50th (median), and 75th quantiles, as well as the minimum and maximum values. Internal-CRCII, internal colorectal cancer stage II cohort; External-CRCII-1, external colorectal cancer stage II cohort 1; External-CRCII-2, external colorectal cancer stage II cohort 2; TCGA-CRCII, TCGA colorectal cancer stage II cohort. (DOCX) [file pmed.1004614.s015.docx]

**S15 Fig. Similarity assessment before and after heatmap processing.**

Box plots illustrating similarities before and after heatmap processing in Internal-CRCII (a), External-CRCII-1 (b), External-CRCII-2 (c), and TCGA-CRCII (d). Similarity was measured using the Structural Similarity Index Method (SSIM). The plots display the 25th, 50th (median), and 75th quantiles, as well as the minimum and maximum values. Internal-CRCII, internal colorectal cancer stage II cohort; External-CRCII-1, external colorectal cancer stage II cohort 1; External-CRCII-2, external colorectal cancer stage II cohort 2; TCGA-CRCII, TCGA colorectal cancer stage II cohort.
