# Supplementary material for: Multiview deep-learning-enabled histopathology for prognostic and therapeutic stratification in stage II colorectal cancer: A retrospective multicenter study
Source: PLoS Med. 2026 Jan 13;23(1):e1004614. doi: 10.1371/journal.pmed.1004614 (PMC12801286; doi:10.1371/journal.pmed.1004614)
Supplement: S16 Fig — (a–d) Frequency distribution histograms of TLS area in Internal-CRCII (a), External-CRCII-1 (b), External-CRCII-2 (c), and TCGA-CRCII (d). (e–h) Frequency distribution histograms of the distance between TLS and tumor margin in Internal-CRCII (e), External-CRCII-1 (f), External-CRCII-2 (g), and TCGA-CRCII (h). (i–l) Bar graphs showing TLS subtypes in Internal-CRCII (i), External-CRCII-1 (j), External-CRCII-2 (k), and TCGA-CRCII (l). TLS, tertiary lymphoid structure; Agg, aggregates; FL-1, primary follicles; FL-2, secondary follicles; Internal-CRCII, internal colorectal cancer stage II cohort; External-CRCII-1, external colorectal cancer stage II cohort 1; External-CRCII-2, external colorectal cancer stage II cohort 2; TCGA-CRCII, TCGA colorectal cancer stage II cohort. (DOCX) [file pmed.1004614.s016.docx]

**S16 Fig. Statistical analysis of TLS spatial characteristics.**

(a-d) Frequency distribution histograms of TLS area in Internal-CRCII (a), External-CRCII-1 (b), External-CRCII-2 (c), and TCGA-CRCII (d). (e-h) Frequency distribution histograms of the distance between TLS and tumor margin in Internal-CRCII (e), External-CRCII-1 (f), External-CRCII-2 (g), and TCGA-CRCII (h). (i-l) Bar graphs showing TLS subtypes in Internal-CRCII (i), External-CRCII-1 (j), External-CRCII-2 (k), and TCGA-CRCII (l). TLS, tertiary lymphoid structure; Agg, aggregates; FL-1, primary follicles; FL-2, secondary follicles; Internal-CRCII, internal colorectal cancer stage II cohort; External-CRCII-1, external colorectal cancer stage II cohort 1; External-CRCII-2, external colorectal cancer stage II cohort 2; TCGA-CRCII, TCGA colorectal cancer stage II cohort.
