# Supplementary material for: Multiview deep-learning-enabled histopathology for prognostic and therapeutic stratification in stage II colorectal cancer: A retrospective multicenter study
Source: PLoS Med. 2026 Jan 13;23(1):e1004614. doi: 10.1371/journal.pmed.1004614 (PMC12801286; doi:10.1371/journal.pmed.1004614)
Supplement: S1 Table — TLS, tertiary lymphoid structure; Internal-CRCII, internal colorectal cancer stage II cohort; External-CRCII-1, external colorectal cancer stage II cohort 1; External-CRCII-2, external colorectal cancer stage II cohort 2. (DOCX) [file pmed.1004614.s017.docx]

**S1 Table. Classification performance of TNTM.**

| Cohorts | Category | Predictive performance | | |
| --- | --- | --- | --- | --- |
|  |  | Sensitivity (95%CI) | Specificity (95%CI) | AUROC (95%CI) |
| Internal-CRCII | TLS-like | 0.9914 (0.9907, 0.992) | 0.9941 (0.9937, 0.9945) | 0.9997 (0.9997, 0.9997) |
|  | Normal | 0.9835 (0.9826, 0.9843) | 0.9908 (0.9902, 0.9913) | 0.9991 (0.9991, 0.9992) |
|  | Tumor | 0.9887 (0.9878, 0.9896) | 0.996 (0.9956, 0.9963) | 0.9997 (0.9996, 0.9997) |
| External-CRCII-1 | TLS-like | 0.9854 (0.9841, 0.9867) | 0.9952 (0.9945, 0.9958) | 0.9997 (0.9997, 0.9997) |
|  | Normal | 0.9993 (0.9988, 0.9996) | 0.9524 (0.9506, 0.9542) | 0.9995 (0.9994, 0.9996) |
|  | Tumor | 0.8885 (0.8841, 0.8927) | 0.9996 (0.9994, 0.9997) | 0.9993 (0.9992, 0.9994) |
| External-CRCII-2 | TLS-like | 0.8992 (0.8969, 0.9016) | 0.996 (0.9958, 0.9962) | 0.9979 (0.9978, 0.998) |
|  | Normal | 0.9962 (0.996, 0.9965) | 0.8976 (0.8964, 0.8987) | 0.9966 (0.9965, 0.9968) |
|  | Tumor | 0.8895 (0.8881, 0.8909) | 0.9977 (0.975, 0.9978) | 0.9963 (0.9962, 0.9964) |

TLS, tertiary lymphoid structure; Internal-CRCII, internal colorectal cancer stage II cohort; External-CRCII-1, external colorectal cancer stage II cohort 1; External-CRCII-2, external colorectal cancer stage II cohort 2.
