# Supplementary material for: Multiview deep-learning-enabled histopathology for prognostic and therapeutic stratification in stage II colorectal cancer: A retrospective multicenter study
Source: PLoS Med. 2026 Jan 13;23(1):e1004614. doi: 10.1371/journal.pmed.1004614 (PMC12801286; doi:10.1371/journal.pmed.1004614)
Supplement: S2 Table — Agg, aggregates; FL-1, primary follicles; FL-2, secondary follicles; Internal-CRCII, internal colorectal cancer stage II cohort; External-CRCII-1, external colorectal cancer stage II cohort 1; External-CRCII-2, external colorectal cancer stage II cohort 2. (DOCX) [file pmed.1004614.s018.docx]

**S2 Table. Classification performance of TLSM.**

| Cohorts | Category | Predictive performance | | |
| --- | --- | --- | --- | --- |
|  |  | Sensitivity (95%CI) | Specificity (95%CI) | AUROC (95%CI) |
| Internal-CRCII | Agg | 0.8493 (0.8132, 0.8809) | 0.8876 (0.863, 0.9091) | 0.9476 (0.9366, 0.9586) |
|  | FL-1 | 0.7905 (0.7514, 0.826) | 0.8677 (0.8412, 0.8913) | 0.9100 (0.8941, 0.9259) |
|  | FL-2 | 0.8799 (0.8362, 0.9153) | 0.9787 (0.9673, 0.987) | 0.9851 (0.9787, 0.9916) |
| External-CRCII-1 | Agg | 0.9621 (0.9446, 0.9753) | 0.8845 (0.8517, 0.9123) | 0.9781 (0.9705, 0.9856) |
|  | FL-1 | 0.8282 (0.7828, 0.8676) | 0.9672 (0.952,3 0.9785) | 0.9641 (0.9526, 0.9755) |
|  | FL-2 | 0.9624 (0.9144, 0.9877) | 0.9929 (0.9854, 0.9971) | 0.9982 (0.9967, 0.9996) |
| External-CRCII-2 | Agg | 0.8884 (0.8681, 0.9065) | 0.9357 (0.9121, 0.9546) | 0.9622 (0.9527, 0.9718) |
|  | FL-1 | 0.8759 (0.8411, 0.9054) | 0.8958 (0.8772, 0.9124) | 0.9379 (0.9248, 0.951) |
|  | FL-2 | 0.936 (0.8778, 0.972) | 0.9862 (0.9789, 0.9914) | 0.9923 (0.9872, 0.9975) |

Agg, aggregates; FL-1, primary follicles; FL-2, secondary follicles; Internal-CRCII, internal colorectal cancer stage II cohort; External-CRCII-1, external colorectal cancer stage II cohort 1; External-CRCII-2, external colorectal cancer stage II cohort 2.
