# Supplementary material for: Multiview deep-learning-enabled histopathology for prognostic and therapeutic stratification in stage II colorectal cancer: A retrospective multicenter study
Source: PLoS Med. 2026 Jan 13;23(1):e1004614. doi: 10.1371/journal.pmed.1004614 (PMC12801286; doi:10.1371/journal.pmed.1004614)
Supplement: S3 Table — Internal-CRCII, internal colorectal cancer stage II cohort; External-CRCII-1, external colorectal cancer stage II cohort 1; External-CRCII-2, external colorectal cancer stage II cohort 2; TCGA-CRCII, TCGA colorectal cancer stage II cohort. (DOCX) [file pmed.1004614.s019.docx]

**S3 Table. Sensitivity and specificity of MVNet at various thresholds across four datasets.**

|  |  | Dataset | | | |
| --- | --- | --- | --- | --- | --- |
| Threshold | Metrics | Internal-CRCII | External-CRCII-1 | External-CRCII-2 | TCGA-CRCII |
| 0.01 | Sensitivity | 0.773 | 0.567 | 0.817 | 0.970 |
|  | Specificity | 0.702 | 0.796 | 0.594 | 0.250 |
|  | Significance | ****p<0.0001 | ****p<0.0001 | ****p<0.0001 | **p=0.0088 |
| 0.1 | Sensitivity | 0.647 | 0.463 | 0.700 | 0.848 |
|  | Specificity | 0.843 | 0.895 | 0.712 | 0.452 |
|  | Significance | ****p<0.0001 | ****p<0.0001 | ****p<0.0001 | **p=0.001 |
| 0.2 | Sensitivity | 0.571 | 0.403 | 0.533 | 0.788 |
|  | Specificity | 0.877 | 0.940 | 0.801 | 0.488 |
|  | Significance | ****p<0.0001 | ****p<0.0001 | ****p<0.0001 | **p=0.0047 |
| 0.3 | Sensitivity | 0.521 | 0.373 | 0.467 | 0.788 |
|  | Specificity | 0.897 | 0.954 | 0.852 | 0.500 |
|  | Significance | ****p<0.0001 | ****p<0.0001 | ****p<0.0001 | **p=0.0023 |
| 0.4 | Sensitivity | 0.487 | 0.343 | 0.467 | 0.727 |
|  | Specificity | 0.904 | 0.961 | 0.882 | 0.560 |
|  | Significance | ****p<0.0001 | ****p<0.0001 | ****p<0.0001 | ***p=0.0008 |
| 0.5 | Sensitivity | 0.454 | 0.343 | 0.417 | 0.697 |
|  | Specificity | 0.917 | 0.965 | 0.919 | 0.595 |
|  | Significance | ****p<0.0001 | ****p<0.0001 | ****p<0.0001 | ***p=0.0006 |
| EER | Threshold | 0.075 | 0.006 | 0.090 | 0.584 |
|  | Sensitivity | 0.748 | 0.716 | 0.700 | 0.667 |
|  | Specificity | 0.756 | 0.716 | 0.701 | 0.619 |
|  | Significance | ****p<0.0001 | ****p<0.0001 | ****p<0.0001 | ***p=0.00099 |

Internal-CRCII, internal colorectal cancer stage II cohort; External-CRCII-1, external colorectal cancer stage II cohort 1; External-CRCII-2, external colorectal cancer stage II cohort 2; TCGA-CRCII, TCGA colorectal cancer stage II cohort.
