# Supplementary material for: Multiview deep-learning-enabled histopathology for prognostic and therapeutic stratification in stage II colorectal cancer: A retrospective multicenter study
Source: PLoS Med. 2026 Jan 13;23(1):e1004614. doi: 10.1371/journal.pmed.1004614 (PMC12801286; doi:10.1371/journal.pmed.1004614)
Supplement: S4 Table — ACT, adjuvant chemotherapy; Internal-CRCII, internal colorectal cancer stage II cohort; External-CRCII-1, external colorectal cancer stage II cohort 1; External-CRCII-2, external colorectal cancer stage II cohort 2; TCGA-CRCII, TCGA colorectal cancer stage II cohort. (DOCX) [file pmed.1004614.s020.docx]

**S4 Table. Univariable Cox regression analysis of MVNet-predicted risk in adjuvant chemotherapy (ACT) positive and negative subgroups across multiple cohorts.**

| Dataset | Group | Pvalue | HR |
| --- | --- | --- | --- |
| Internal-CRCII | ACT+ | < 0.001 | 7.79 (3.82, 15.9) |
|  | ACT- | < 0.001 | 10.26 (6.29, 16.73) |
| External-CRCII-1 | ACT+ | 0.001 | 7.42 (2.38, 23.1) |
|  | ACT- | < 0.001 | 18.12 (8.06, 40.73) |
| External-CRCII-2 | ACT+ | < 0.001 | 5.95 (2.25, 15.76) |
|  | ACT- | < 0.001 | 14.63 (6.76, 31.63) |

ACT, adjuvant chemotherapy; Internal-CRCII, internal colorectal cancer stage II cohort; External-CRCII-1, external colorectal cancer stage II cohort 1; External-CRCII-2, external colorectal cancer stage II cohort 2; TCGA-CRCII, TCGA colorectal cancer stage II cohort.
