# Supplementary material for: Multiview deep-learning-enabled histopathology for prognostic and therapeutic stratification in stage II colorectal cancer: A retrospective multicenter study
Source: PLoS Med. 2026 Jan 13;23(1):e1004614. doi: 10.1371/journal.pmed.1004614 (PMC12801286; doi:10.1371/journal.pmed.1004614)
Supplement: S5 Table — MVNet, multi-view network; PNI, perineural invasion; VI, vascular invasion; SRCC, signet-ring cell carcinoma; MAC, mucinous adenocarcinoma; LNS, lymph node sampling; MMR, mismatch repair; ACT, adjuvant chemotherapy; BD3, tumor budding grade 1–3; Internal-CRCII, internal colorectal cancer stage II cohort; External-CRCII-1, external colorectal cancer stage II cohort 1; External-CRCII-2, external colorectal cancer stage II cohort 2. (DOCX) [file pmed.1004614.s021.docx]

**S5 Table. High-Risk Feature Distribution by MVNet Risk Group and ACT Status.**

| Dataset | Risk group | ACT | N | High-risk clinicopathological features | | | | | | | | | | | |
| --- | --- | --- | --- | --- | --- | --- | --- | --- | --- | --- | --- | --- | --- | --- | --- |
|  |  |  |  | T4 | LNS<12 | pMMR | CEA≥5 | CA199≥37 | VI | PI | BD3 | Perforation | High grading | MAC | SRCC |
| Internal-  CRCII | High risk | Positive | 108 | 25 | 28 | 98 | 39 | 14 | 10 | 29 | 3 | 13 | 35 | 17 | 2 |
|  |  | Negative | 149 | 23 | 30 | 139 | 67 | 26 | 14 | 46 | 6 | 10 | 57 | 29 | 1 |
|  | Low risk | Positive | 190 | 59 | 20 | 172 | 68 | 28 | 23 | 62 | 11 | 23 | 60 | 48 | 2 |
|  |  | Negative | 334 | 46 | 32 | 261 | 114 | 44 | 18 | 53 | 16 | 53 | 107 | 73 | 3 |
|  | Overall | Positive | 298 | 84 | 48 | 270 | 107 | 42 | 33 | 91 | 14 | 36 | 95 | 65 | 4 |
|  |  | Negative | 483 | 69 | 62 | 400 | 181 | 70 | 32 | 99 | 22 | 63 | 164 | 102 | 4 |
| External-CRCII-1 | High risk | Positive | 64 | 17 | 25 | 63 | 29 | 9 | 0 | 11 | 3 | 4 | 12 | 5 | 0 |
|  |  | Negative | 63 | 17 | 17 | 63 | 32 | 9 | 2 | 14 | 3 | 3 | 11 | 15 | 0 |
|  | Low risk | Positive | 95 | 16 | 25 | 94 | 40 | 7 | 2 | 14 | 6 | 11 | 11 | 15 | 1 |
|  |  | Negative | 130 | 25 | 24 | 127 | 60 | 15 | 3 | 19 | 5 | 12 | 17 | 19 | 1 |
|  | Overall | Positive | 159 | 33 | 50 | 157 | 69 | 16 | 2 | 25 | 9 | 15 | 23 | 20 | 1 |
|  |  | Negative | 193 | 42 | 41 | 190 | 92 | 24 | 5 | 33 | 8 | 15 | 28 | 34 | 1 |
| External-CRCII-2 | High risk | Positive | 64 | 20 | 9 | 62 | 26 | 12 | 2 | 9 | 6 | 5 | 9 | 5 | 0 |
|  |  | Negative | 62 | 15 | 13 | 59 | 26 | 12 | 3 | 16 | 7 | 2 | 13 | 9 | 1 |
|  | Low risk | Positive | 109 | 31 | 15 | 93 | 50 | 15 | 5 | 10 | 3 | 23 | 28 | 13 | 1 |
|  |  | Negative | 107 | 36 | 13 | 92 | 53 | 17 | 2 | 12 | 2 | 16 | 25 | 18 | 0 |
|  | Overall | Positive | 173 | 51 | 24 | 155 | 76 | 27 | 7 | 19 | 9 | 28 | 37 | 18 | 1 |
|  |  | Negative | 169 | 51 | 26 | 151 | 79 | 29 | 5 | 28 | 9 | 18 | 38 | 27 | 1 |

MVNet, multi-view network; PNI, perineural invasion; VI, vascular invasion; SRCC, signet-ring cell carcinoma; MAC, mucinous adenocarcinoma; LNS, lymph node sampling; MMR, mismatch repair; ACT, adjuvant chemotherapy; BD3, tumor budding grade 1-3; Internal-CRCII, internal colorectal cancer stage II cohort; External-CRCII-1, external colorectal cancer stage II cohort 1; External-CRCII-2, external colorectal cancer stage II cohort 2.
