# Supplementary material for: Multiview deep-learning-enabled histopathology for prognostic and therapeutic stratification in stage II colorectal cancer: A retrospective multicenter study
Source: PLoS Med. 2026 Jan 13;23(1):e1004614. doi: 10.1371/journal.pmed.1004614 (PMC12801286; doi:10.1371/journal.pmed.1004614)
Supplement: S6 Table — MVNet, multi-view network; ACT, adjuvant chemotherapy; Internal-CRCII, internal colorectal cancer stage II cohort; External-CRCII-1, external colorectal cancer stage II cohort 1; External-CRCII-2, external colorectal cancer stage II cohort 2. (DOCX) [file pmed.1004614.s022.docx]

**S6 Table Interaction analysis between ACT and MVNet risk group using Cox proportional hazards models across cohorts.**

| Dataset | Variable | HR (95% CI) | p-value |
| --- | --- | --- | --- |
| Internal-CRCII | ACT (chemo) | 0.77 (0.35–1.70) | 0.518 |
|  | MVNet high risk (strat1) | 8.61 (5.20–14.28) | <0.001 *** |
|  | Interaction chemo:strat1 | 0.77 (0.31–1.90) | 0.572 |
| External-CRCII-1 | ACT (chemo) | 1.55 (0.64–3.75) | 0.328 |
|  | MVNet high risk (strat1) | 8.30 (3.93–17.56) | <0.001 *** |
|  | Interaction chemo:strat1 | 0.35 (0.12–0.98) | 0.046 * |
| External-CRCII-2 | ACT (chemo) | 1.00 (0.39–2.49) | 0.979 |
|  | MVNet high risk (strat1) | 7.64 (3.61–16.16) | <0.001 *** |
|  | Interaction chemo:strat1 | 0.35 (0.11–1.05) | 0.062 |

MVNet, multi-view network; ACT, adjuvant chemotherapy; Internal-CRCII, internal colorectal cancer stage II cohort; External-CRCII-1, external colorectal cancer stage II cohort 1; External-CRCII-2, external colorectal cancer stage II cohort 2.
