# Supplementary material for: Multiview deep-learning-enabled histopathology for prognostic and therapeutic stratification in stage II colorectal cancer: A retrospective multicenter study
Source: PLoS Med. 2026 Jan 13;23(1):e1004614. doi: 10.1371/journal.pmed.1004614 (PMC12801286; doi:10.1371/journal.pmed.1004614)
Supplement: S7 Table — MVNet, multi-view network; PNI, perineural invasion; VI, vascular invasion; SRCC, signet-ring cell carcinoma; MAC, mucinous adenocarcinoma; LNS, lymph node sampling; MMR, mismatch repair. (DOCX) [file pmed.1004614.s023.docx]

**S7 Table. List of 14 key clinicopathological parameters used for comparison with MVNet in this study.**

| **Parameter** |
| --- |
| Differentiation |
| Perforation |
| PNI |
| VI |
| SRCC |
| MAC |
| Classification |
| Primary site |
| LNS |
| T stage |
| CA199 |
| CEA |
| Tumor budding |
| MMR |

MVNet, multi-view network; PNI, perineural invasion; VI, vascular invasion; SRCC, signet-ring cell carcinoma; MAC, mucinous adenocarcinoma; LNS, lymph node sampling; MMR, mismatch repair.
