# Supplementary material for: Multiview deep-learning-enabled histopathology for prognostic and therapeutic stratification in stage II colorectal cancer: A retrospective multicenter study
Source: PLoS Med. 2026 Jan 13;23(1):e1004614. doi: 10.1371/journal.pmed.1004614 (PMC12801286; doi:10.1371/journal.pmed.1004614)
Supplement: S8 Table — MVNet, multi-view network. (DOCX) [file pmed.1004614.s024.docx]

**S8 Table. Comparison of prognostic performance of MVNet across different numbers of randomly sampled tumor slides per patient in a subset of 50 patients.**

| Slide count | AUROC | 95% CI |
| --- | --- | --- |
| 1 | 0.789 | (0.647, 0.909) |
| 2 | 0.798 | (0.670, 0.911) |
| 3 | 0.806 | (0.670, 0.915) |
| 4 | 0.830 | (0.707, 0.937) |
| All | 0.840 | (0.727, 0.935) |

MVNet, multi-view network.
