# Supplementary material for: Multiview deep-learning-enabled histopathology for prognostic and therapeutic stratification in stage II colorectal cancer: A retrospective multicenter study
Source: PLoS Med. 2026 Jan 13;23(1):e1004614. doi: 10.1371/journal.pmed.1004614 (PMC12801286; doi:10.1371/journal.pmed.1004614)
Supplement: S9 Table — TLS, tertiary lymphoid structure. (DOCX) [file pmed.1004614.s025.docx]

**S9 Table. Summary of 226 TLS-related features used in SpB input.**

| Feature Type | Dimensions or Categories | Feature Count | Description |
| --- | --- | --- | --- |
| Count of TLS | 7 distances × 7 areas × 3 TLS categories | 147 | Number of TLSs per sub-category |
| TLS count and area | 7 distances × 3 TLS categories × 2 types (count + area) | 42 | Total count and area of TLSs in each distance-category and TLS category |
| TLS count and area | (7 distances + 7 areas + 3 categories) × 2 (count + area) | 34 | Total count and area in each dimension |
| Global metrics | - | 3 | Total TLS area, count, and tumor area in the whole WSIs |
| Total | - | 226 | All quantitative features were normalized by the number of slides for each individual patient |

TLS, tertiary lymphoid structure.
