# Supplementary material for: Multiview deep-learning-enabled histopathology for prognostic and therapeutic stratification in stage II colorectal cancer: A retrospective multicenter study
Source: PLoS Med. 2026 Jan 13;23(1):e1004614. doi: 10.1371/journal.pmed.1004614 (PMC12801286; doi:10.1371/journal.pmed.1004614)
Supplement: S1 Checklist — This checklist is made available under the Creative Commons Attribution 4.0 (CC BY 4.0) license. The original STROBE checklist can be accessed at https://www.strobe-statement.org/. (DOCX) [file pmed.1004614.s026.docx]

STROBE Statement—Checklist of items that should be included in reports of ***cohort studies***

|  | Item No | Recommendation | Page No |
| --- | --- | --- | --- |
| **Title and abstract** | 1 | (*a*) Indicate the study’s design with a commonly used term in the title or the abstract | Abstract |
|  |  | (*b*) Provide in the abstract an informative and balanced summary of what was done and what was found | Abstract |
| Introduction | | | |
| Background/rationale | 2 | Explain the scientific background and rationale for the investigation being reported | Introduction, first paragraph |
| Objectives | 3 | State specific objectives, including any prespecified hypotheses | Introduction, fourth paragraph |
| Methods | | | |
| Study design | 4 | Present key elements of study design early in the paper | Methods, second paragraph |
| Setting | 5 | Describe the setting, locations, and relevant dates, including periods of recruitment, exposure, follow-up, and data collection | Methods, third paragraph |
| Participants | 6 | (*a*) Give the eligibility criteria, and the sources and methods of selection of participants. Describe methods of follow-up | Methods, fourth paragraph |
|  |  | (*b*) For matched studies, give matching criteria and number of exposed and unexposed | Not applicable |
| Variables | 7 | Clearly define all outcomes, exposures, predictors, potential confounders, and effect modifiers. Give diagnostic criteria, if applicable | Methods, fourth paragraph |
| Data sources/ measurement | 8* | For each variable of interest, give sources of data and details of methods of assessment (measurement). Describe comparability of assessment methods if there is more than one group | Methods, third paragraph |
| Bias | 9 | Describe any efforts to address potential sources of bias | Methods, third paragraph |
| Study size | 10 | Explain how the study size was arrived at | Methods, fourth paragraph |
| Quantitative variables | 11 | Explain how quantitative variables were handled in the analyses. If applicable, describe which groupings were chosen and why | Methods, statistical analysis |
| Statistical methods | 12 | (*a*) Describe all statistical methods, including those used to control for confounding | Methods, statistical analysis |
|  |  | (*b*) Describe any methods used to examine subgroups and interactions | Methods, statistical analysis |
|  |  | (*c*) Explain how missing data were addressed | Methods, fourth paragraph |
|  |  | (*d*) If applicable, explain how loss to follow-up was addressed | Methods, fourth paragraph |
|  |  | (*e*) Describe any sensitivity analyses | Methods, training and evaluation |
| Results | | |  |
| Participants | 13* | (a) Report numbers of individuals at each stage of study—eg numbers potentially eligible, examined for eligibility, confirmed eligible, included in the study, completing follow-up, and analysed | Results, first paragraph |
|  |  | (b) Give reasons for non-participation at each stage | Results, first paragraph |
|  |  | (c) Consider use of a flow diagram | Results, first paragraph |
| Descriptive data | 14* | (a) Give characteristics of study participants (eg demographic, clinical, social) and information on exposures and potential confounders | Results, first paragraph |
|  |  | (b) Indicate number of participants with missing data for each variable of interest | Results, first paragraph |
|  |  | (c) Summarise follow-up time (eg, average and total amount) | Results, first paragraph |
| Outcome data | 15* | Report numbers of outcome events or summary measures over time | Results, first paragraph |

| Main results | 16 | (*a*) Give unadjusted estimates and, if applicable, confounder-adjusted estimates and their precision (eg, 95% confidence interval). Make clear which confounders were adjusted for and why they were included | Results, section 5 |
| --- | --- | --- | --- |
|  |  | (*b*) Report category boundaries when continuous variables were categorized | Results, section 5 |
|  |  | (*c*) If relevant, consider translating estimates of relative risk into absolute risk for a meaningful time period | Not reported |
| Other analyses | 17 | Report other analyses done—eg analyses of subgroups and interactions, and sensitivity analyses | Results, section 6 |
| Discussion | | | |
| Key results | 18 | Summarise key results with reference to study objectives | Discussion, first paragraph |
| Limitations | 19 | Discuss limitations of the study, taking into account sources of potential bias or imprecision. Discuss both direction and magnitude of any potential bias | Discussion, fifth paragraph |
| Interpretation | 20 | Give a cautious overall interpretation of results considering objectives, limitations, multiplicity of analyses, results from similar studies, and other relevant evidence | Discussion, second paragraph |
| Generalisability | 21 | Discuss the generalisability (external validity) of the study results | Discussion, second paragraph |
| Other information | | | |
| Funding | 22 | Give the source of funding and the role of the funders for the present study and, if applicable, for the original study on which the present article is based | End of manuscript |

*Give information separately for exposed and unexposed groups.

**Note:** An Explanation and Elaboration article discusses each checklist item and gives methodological background and published examples of transparent reporting. The STROBE checklist is best used in conjunction with this article (freely available on the Web sites of PLoS Medicine at http://www.plosmedicine.org/, Annals of Internal Medicine at http://www.annals.org/, and Epidemiology at http://www.epidem.com/). Information on the STROBE Initiative is available at http://www.strobe-statement.org.
